# Identification of hub genes associated with adult acute myeloid leukemia progression through weighted gene co-expression network analysis

**DOI:** 10.18632/aging.202493

**Published:** 2021-02-11

**Authors:** Ruiqi Zhu, Wenyi Lin, Liang Tang, Yu Hu

**Affiliations:** 1 Institute of Hematology, Union Hospital, Tongji Medical College, Huazhong University of Science and Technology, Wuhan 430022, China

**Keywords:** AML, hub genes, WGCNA

## Abstract

Acute myeloid leukemia (AML) is a malignancy of hematopoietic stem cells. Although many candidate genes such as *CEBPA*, *FLT3*, *IDH1*, and *IDH2* have been associated with AML initiation and prognosis, the molecular mechanisms underlying this disease remain unclear. In this study, we used a systemic co-expression analysis method, namely weighted gene co-expression network analysis (WGCNA), to identify new candidate genes associated with adult AML progression and prognosis. We identified around 5,138 differentially expressed genes (DEGs) between AML samples (from The Cancer Genome Atlas database) and normal control samples (from the Genotype-Tissue Expression database). WGCNA identified nine co-expression modules with significant differences based on the DEGs. Among modules, the turquoise and blue ones were the most relevant to AML (P-value: turquoise 0, blue 4.64E-77). GO term and KEGG pathway analyses revealed that pathways that are commonly dysregulated in AML were all enriched in the blue and turquoise modules. A total of 15 hub genes were identified to be crucial for AML progression. PIVOT analysis revealed non-coding RNAs, transcriptional factors, and drugs associated with the hub genes. Finally, survival analysis revealed that one of the hub genes, *CEACAM5*, was significantly associated with AML prognosis and could serve as a potential target for AML treatment.

## INTRODUCTION

Acute myeloid leukemia (AML) is a hematopoietic malignancy with high heterogeneity. The incidence of AML is approximately 4.3 per 100,000 per year in the United States according to the Surveillance, Epidemiology, and End Results database. The mortality of patients with AML is still relatively high, despite the application of advanced therapy methods such as intensive chemotherapy, bone marrow transplantation, and targeted therapy. In the past decades, through novel high-throughput sequencing techniques, multiple somatic mutations have been identified as associated with AML initiation or prognosis. For example, *DNMT3A*, *ASXL1*, *IDH1*, and *IDH2* are frequently mutated in patients with AML. These gene mutations are considered to be acquired early in AML. In contrast, mutations in *FLT3*, *RAS*, and *NPM1* are regarded as secondary events in leukemogenesis. Nevertheless, these findings cannot fully explain why and how AML occurs. Hence, bioinformatic analysis is also used to decipher the molecular mechanisms of leukemogenesis and AML progression, which could provide new therapeutic targets for AML treatment.

Various bio-informatic methods have been used to uncover the mechanisms underlying AML progression or to construct prognosis signatures for patient risk stratification [1–3]. Previously, we had also constructed a 4-microRNA signature to predict the prognosis of pediatric and adolescent AML [4]. Weighted gene co-expression network analysis (WGCNA) is a type of bio-informatic method applied to discover the relationship between genes and phenotypes. It provides a comprehensive method to determine the key regulators, crucial pathways, and potential drug targets associated with AML. In this study, we aimed to identify differentially expressed genes (DEGs) between adult patients with AML and healthy control subjects by using information from The Cancer Genome Atlas (TCGA) and Genotype-Tissue Expression (GTEx) database, respectively. Our purpose was to provide a new perspective on genes associated with AML that could be used as potential diagnostic and therapeutic targets.

## RESULTS

### Differentially expressed genes between AML and normal control samples

After screening and filtration of RNA-seq data in TCGA [5] and GTEx [6] databases, a total of 19,148 genes were obtained, of which 5,166 DEGs were identified between AML and whole-blood normal control samples. Fifty-nine additional genes associated with AML from the NCBI GENE and OMIM [7] databases were also included for further co-expression network construction. Subsequently, a total of 5,225 genes were put forth for the WGCNA.

### Co-expression network construction

**Figure 1:**
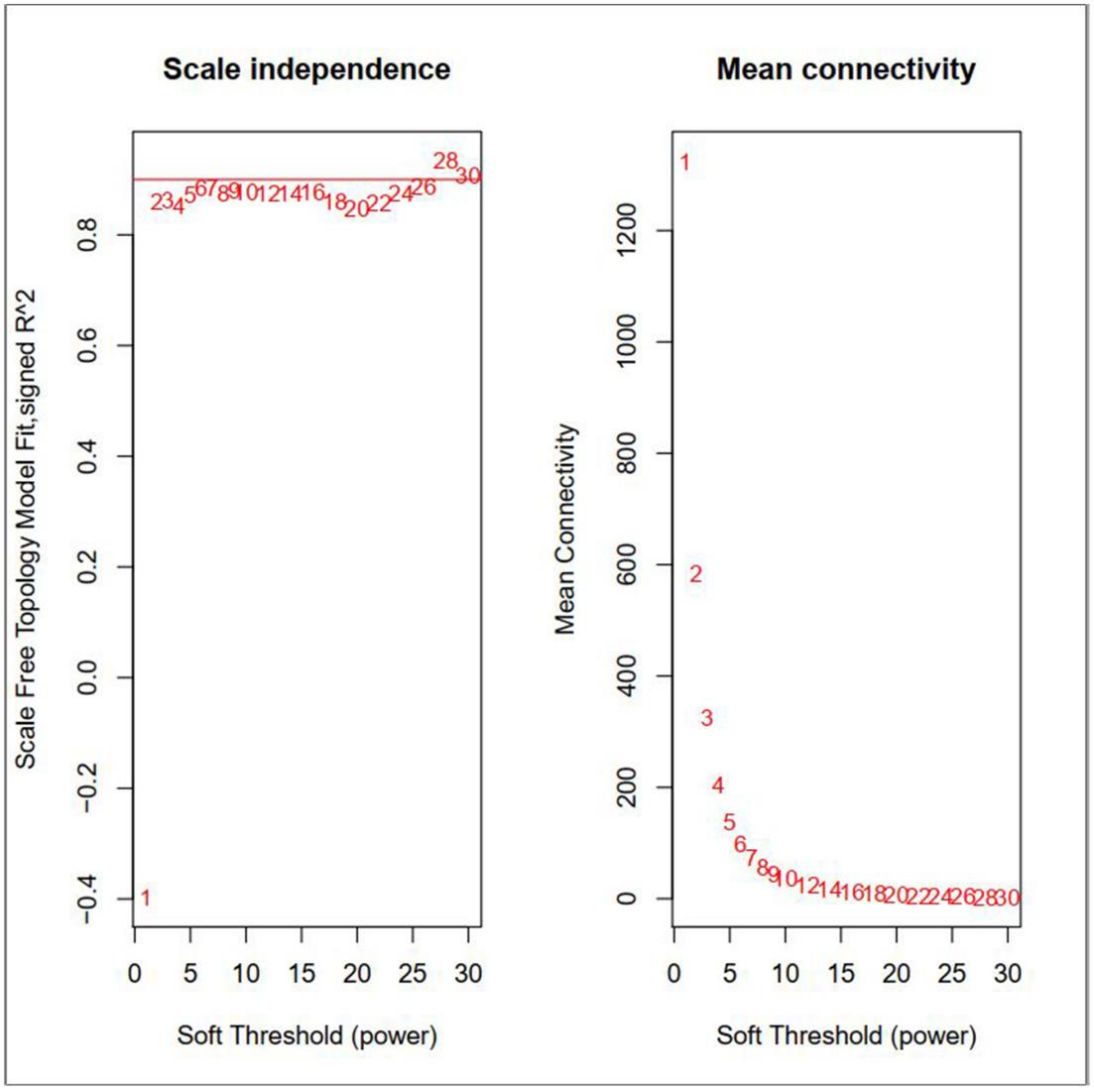
**Effects of different soft-threshold values for adult AML co-expression network.**

**Figure 2:**
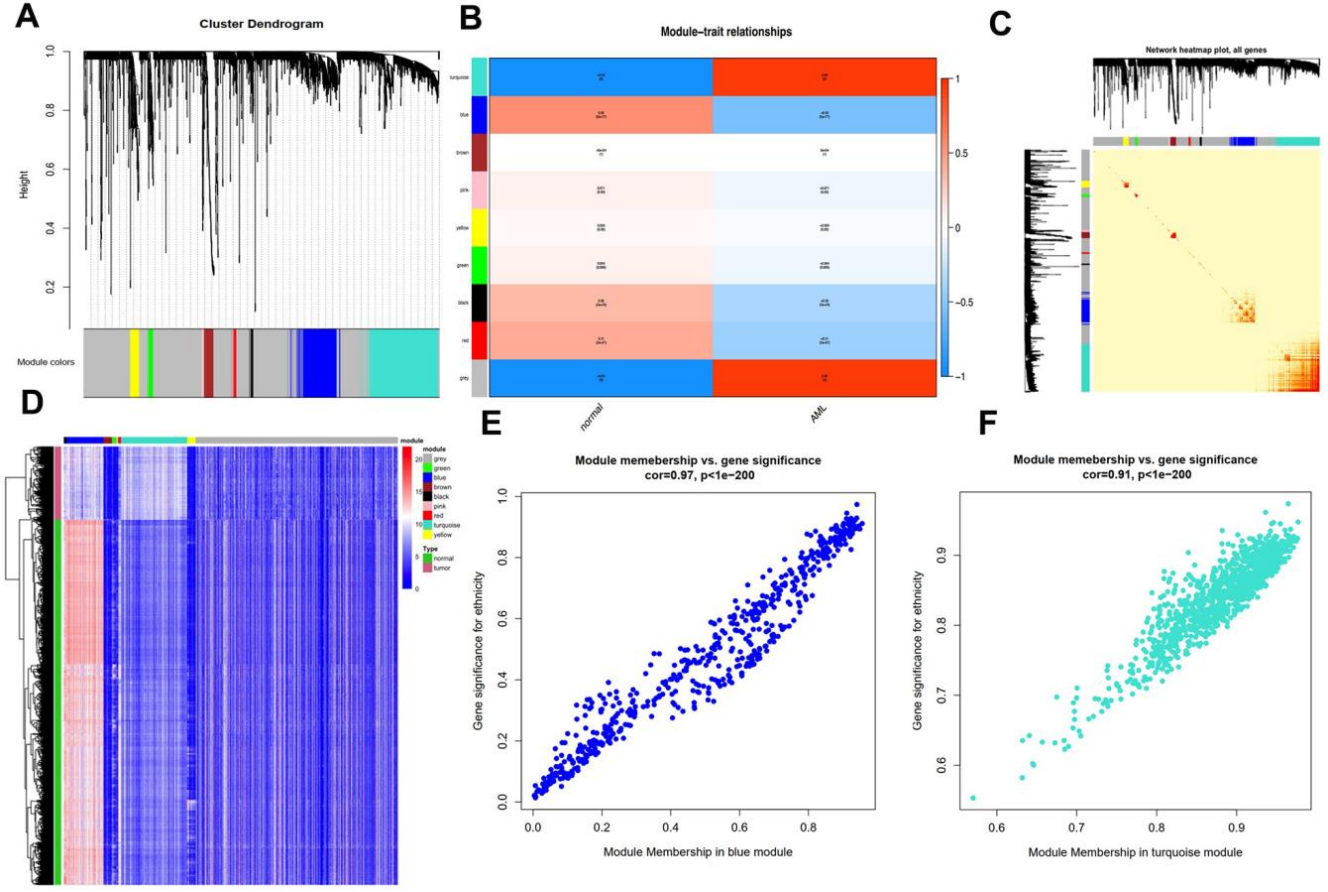
**Characterization of adult AML co-expression network.** (**A**) Cluster dendrogram and colored display of the network. (**B**) Relationship between modules and phenotypes. Turquoise and blue module are the top two modules related with AML phenotype according to P-value. (**C**) Network heatmap plot for genes in the modules in hierarchical clustering dendrograms. The deeper the red, the more correlated between the genes. (**D**) Gene expression differences between AML samples and normal control in modules. (**E**) Gene Significance (GS) and Module Membership (MM) analyses in turquoise and (**F**) blue module.

By carrying out the WGCNA package in R Bioconductor, we constructed a co-expression network for AML. In order to ensure average connectivity and high independence, we screened the power value for the modules, which ranged from 1 to 30. The power value in this study was set at 28 when the scale free R^2^ reached 0.9 at this moment of time (Figure 1). Nine modules were identified, and the number of genes in each module was as follows: 41 in the black, 568 in the blue, 134 in the brown, 66 in the green, 3111 in the gray, 26 in the pink, 45 in the red, 1017 in the turquoise, and 130 in the yellow module. Detailed gene information for each module is provided in Supplementary Table 1. The cluster tree is shown in Figure 2A. Among modules, the turquoise and blue ones were the most relevant for AML (P = 4.64E-77 and 0, respectively; Figure 2B). Network heat-map was used to depict the correlation of genes in and among modules. The depth of the red color correlated with the strength of the relationship between the pairs of modules. As illustrated in Figure 2C, genes within the same module strongly correlated with each other, while genes in different modules were almost independent of each other. This indicated that the modules had great scale independence.

A heat-map was drawn for the modules in 115 AML and 755 control samples. As shown in Figure 2D, genes in the blue module were highly expressed in AML samples, while those in the turquoise module were expressed at lower levels compared to the respective levels in the control samples, suggesting that these two modules were strongly associated with the AML phenotype.

Next, we performed gene significance (GS) and module membership (MM) analyses for genes in the blue and turquoise modules to examine whether they were well correlated with the AML phenotype. As shown in Figure 2E, 2F, genes in both these modules showed strong correlations with the AML phenotype, with P-values < 1e-200.

**Figure 3:**
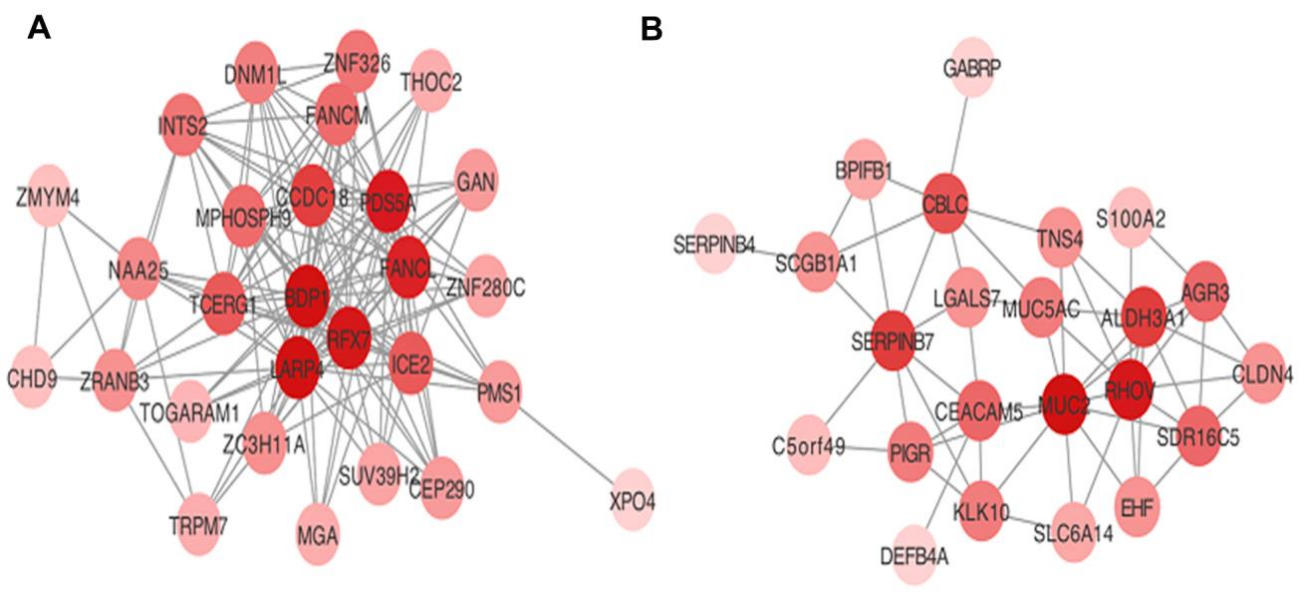
Visualization of hub genes network in (**A**) turquoise and (**B**) blue modules.

Hub genes were considered as genes with connectivity > 5 in both blue and turquoise modules. The 15 hub genes that were identified as associated with AML progression in the present study were: *BDP1*, *RFX7*, *LARP4*, *TCEGR1*, *MPHOSPH9*, *CCDC18*, *PDS5A*, *FANCL*, and *ICE2* in the turquoise module; and *SERPINB7*, *CEACAM5*, *MUC2*, *RHOV*, *ALDH3A1*, and *CBLC* in the blue module. Visualization of the co-expression network for the turquoise and blue modules are depicted in Figure 3.

### GO and KEGG analysis for the turquoise and blue modules

**Figure 4:**
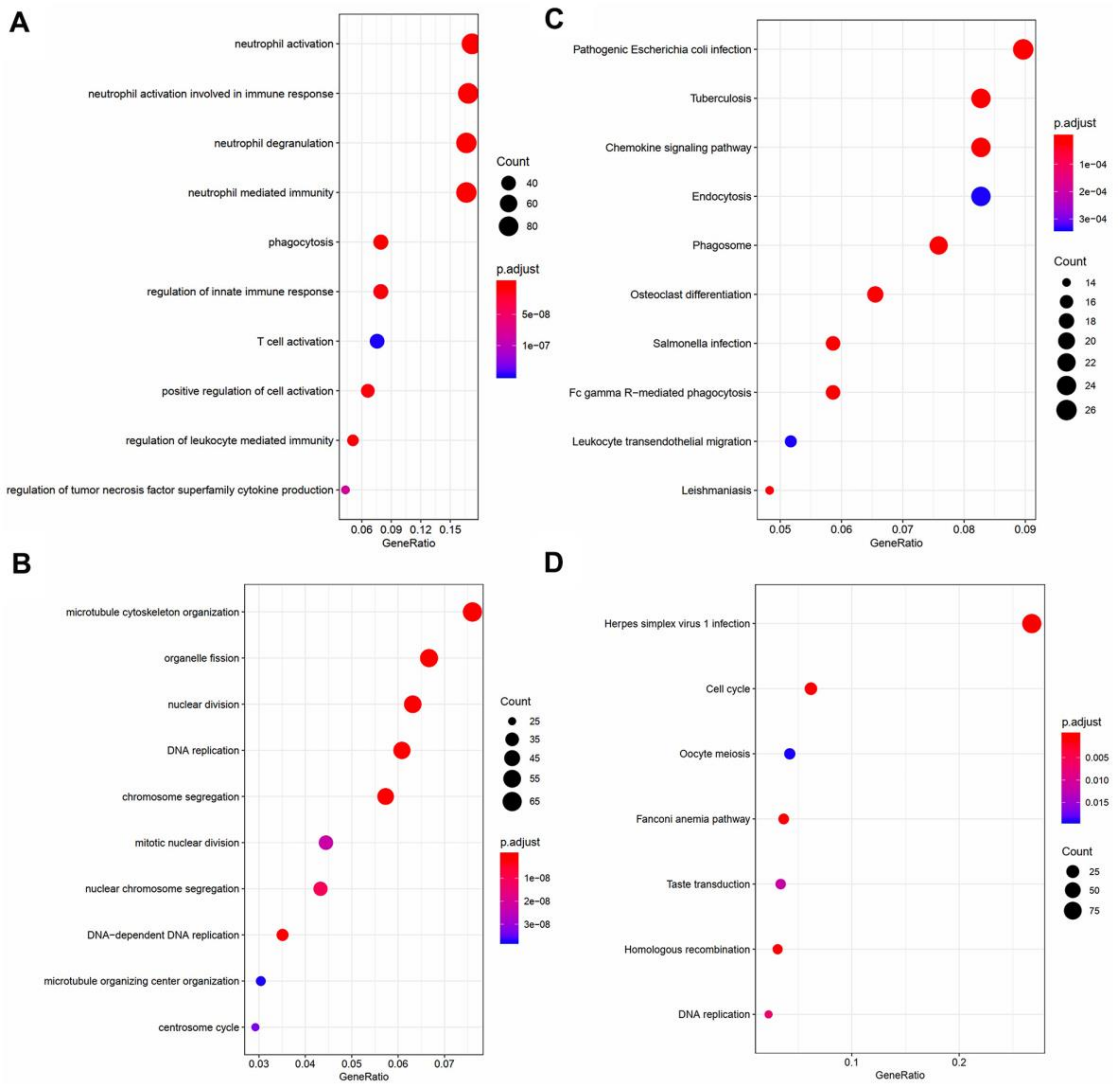
Visualization of part of GO bio-functional analysis results in (**A**) blue and (**C**) turquoise module. Visualization of part of KEGG analysis results in (**B**) blue and (**D**) turquoise module.

In order to obtain a comprehensive understanding of the biological functions of genes in the turquoise and blue modules, we carried out GO and KEGG pathway enrichment analyses. According to GO analysis, genes in the turquoise module were most enriched in DNA replication, chromosome segregation, microtubule cytoskeleton organization, and nuclear division (Figure 4B). In contrast, genes in the blue module were enriched in neutrophil activation, neutrophil degranulation, and neutrophil mediated immunity (Figure 4A). According to KEGG analysis, genes in the turquoise module were most enriched in pathways associated with herpes simplex virus 1 infection, cell cycle, Fanconi anemia pathway, homologous recombination, and DNA replication (Figure 4D), while those in the blue module were enriched in pathways associated with Salmonella infection, phagosome, tuberculosis, and chemokine signaling (Figure 4C). The results of genes in black and red modules were shown in Supplementary Tables 3, 4.

### PIVOT analysis for identifying non-coding RNAs, transcription factors, and drugs associated with AML in the turquoise and blue modules

**Figure 5:**
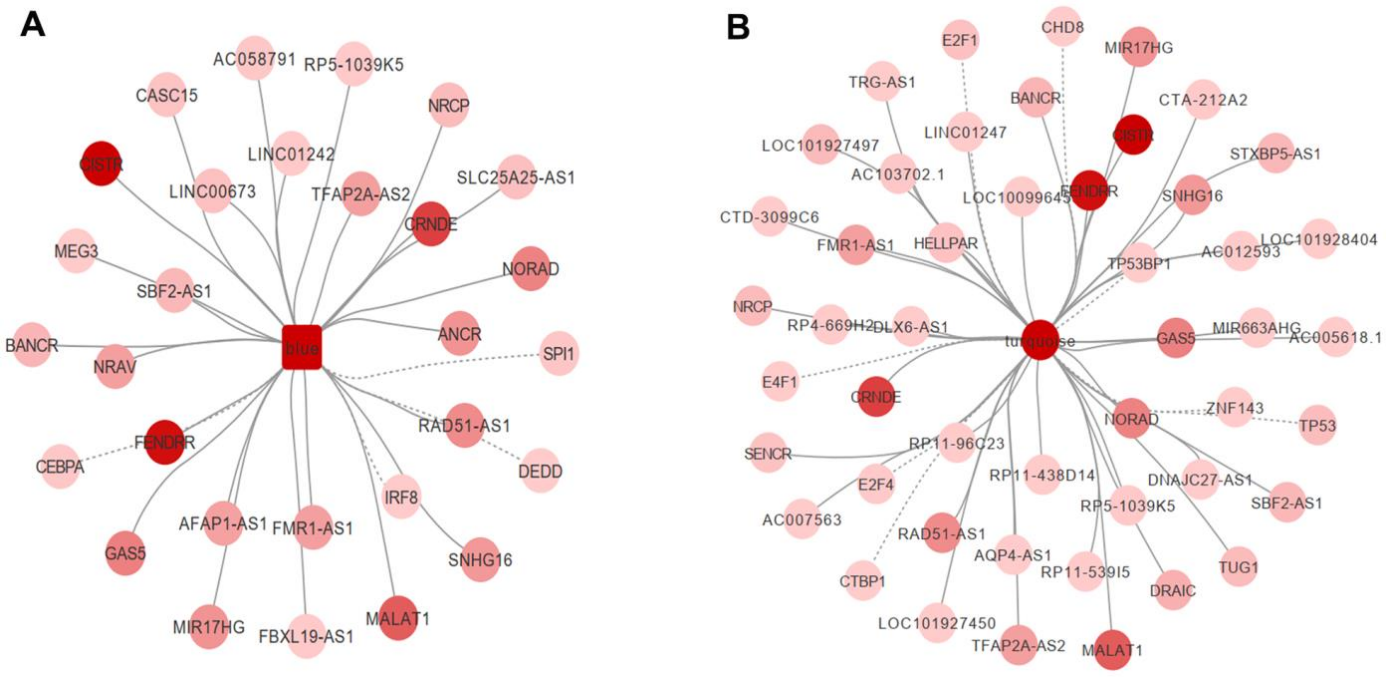
PIVOT analysis revealed TFs and ncRNAs associated with (**A**) blue and (**B**) turquoise module.

Transcription factors are a variety of proteins participating in the initiation of transcription. Mutations or functional dysregulation of transcription factors may result in the transformation of hematopoietic precursors into leukemic stem cells. For example, CCAAT/enhancer binding protein alpha (CEBPA) deficiency inhibits the differentiation of myeloid cells both *in vitro* and *in vivo* [8]. A total of 9,395 transcription factor pairs were enrolled in the present study. PIVOT analysis revealed eight transcription factors (CHD8, CTBP1, E2F1, E2F4, E4F1, TP53, TP53BP1, and ZNF143) in the turquoise module (P < 0.01) and four (CEBPA, DEDD, IRF8, and SPI1) in the blue module as significantly correlated with AML (Figure 5A, 5B).

Non-coding RNAs are important post-transcriptional regulators. Their dysregulation has been tightly correlated to AML progression in a post-transcriptional level. For example, transcriptional activation of some long non-coding RNAs, such as GAS6-AS2, has been reported to lead to chemotherapy resistance in AML [9]. In the present study, 40 and 25 non-coding RNAs in the turquoise and blue module, respectively, were highly associated with AML (Supplementary Table 2 and Figure 5A, 5B). We further investigated drugs associated with hub genes. After screening the DrugBank database, PIVOT analysis identified nine drugs in the blue module [(2S)-2-(3-bromophenyl)-3-(5-chloro-2-hydroxyphenyl)-1,3-thiazolidin-4-one, artenimol, capecitabine, dasatinib, dextromethorphan, interferon-gamma-1b, morniflumate, N-[2-(2-methyl-1H-indol-3-yl)ethyl]thiophene-2-carbox-amide, and sargramostim] and three drugs (caffeine, methionine, and thimerosal) in the turquoise module as significantly associated with AML (P < 0.01 for both).

### Prognostic significance of hub genes in blue and turquoise modules

**Figure 6:**
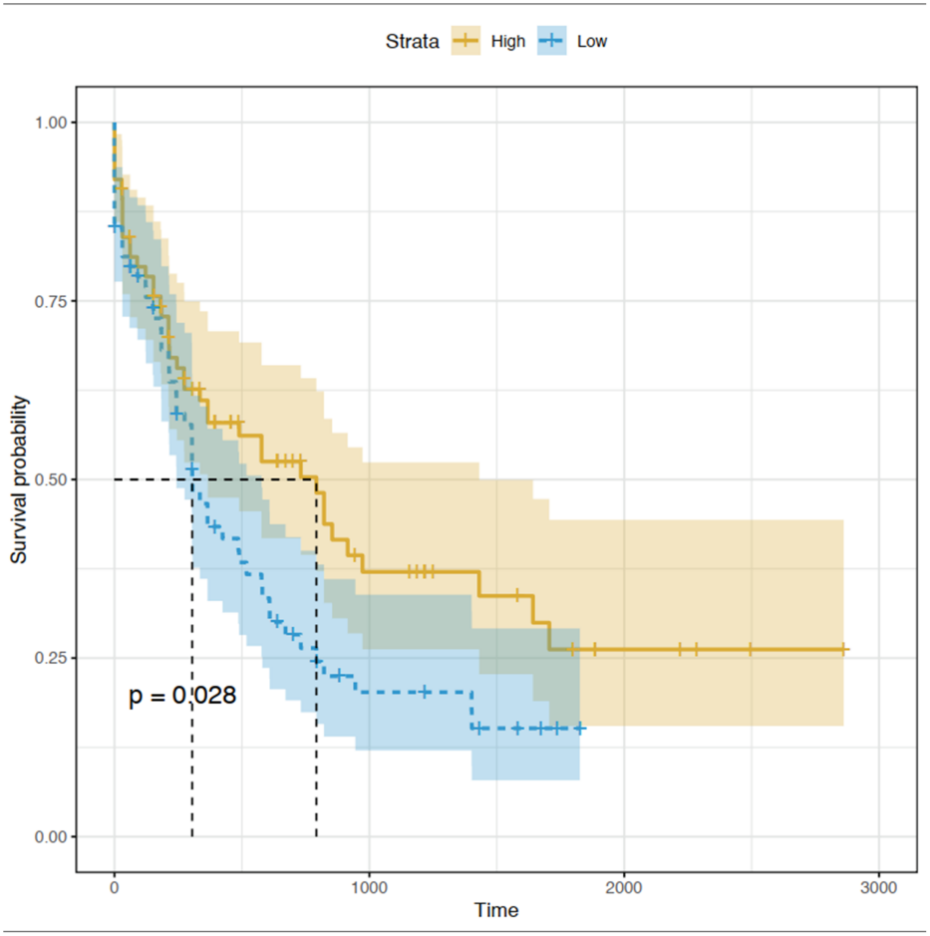
**Kaplan-Meier curve for CEACAM5 low and high expression patients in TCGA database.**

The prognostic value of the 15 identified hub genes was assessed using OS and expression data in TCGA database. We found that high expression of *CEACAM5* was significantly associated with worse OS for patients with AML (Figure 6). Other hub genes were not statistically significantly associated with the survival of these patients.

## DISCUSSION

The molecular mechanisms underlying AML progression and initiation are still not well understood, although advanced progress has been made in the past decades. With the development of high-throughput sequencing technologies, a large amount of genomic data can be acquired from patient samples. In this study, by using published adult AML RNA-seq data from TCGA-AML project and the GTEx database, we constructed a co-expression network by the WGCNA method. A total of nine independent modules were identified, and 15 hub genes were selected from two modules mostly associated with the AML phenotype. Among all hub genes, only *CEACAM5* was significantly associated with the OS of patients with AML, indicating its role as biomarker for AML prognosis and treatment.

WGCNA is a bio-informatic method used to identify clusters of biologically relevant genes associated with a particular disease [10]. It has been widely used to reveal the molecular mechanisms underlying cancer progression and initiation. Bao et al. reported four hub genes (*FOXC1*, *BCL11A*, *FAM171A1*, and *RGMA*) as positively correlated with the triple-negative breast cancer subtype using the WGCNA method [11]. Chen et al. conducted WGCNA and identified the long non-coding RNA LOC646762 as a biomarker for the prognosis of AML in adult patients [12]. According to WGCNA, biologically relevant genes are classified into the same module. In the present study, the turquoise and blue modules were the ones mostly associated with the AML phenotype; hence, genes in these two modules were used to perform GO and KEGG analyses. For the turquoise module, the identified biological processes, i.e., DNA replication [13], chromosome abnormalities [14], and microtubule cytoskeleton organization [15], have been reported to have a great impact on AML progression and drug resistance. For the blue module, the identified biological processes, i.e., neutrophil activation, neutrophil degranulation [16], and neutrophil mediated immunity [17], are all AML oncogene-related processes. Dysregulation of these processes would lead to cell differentiation disorder and ultimately result in AML. Regarding our results of the KEGG pathway analysis, we found that genes in the turquoise and blue modules were highly enriched in cell cycle, homologous recombination [18], DNA replication [19], chemokine signaling, and leukocyte transendothelial migration pathways, which were highly correlated with the AML progression and initiation.

Regarding the identified hub genes, the functions of *BDP1*, *RFX7*, *LARP4*, *TCEGR1*, *MPHOSPH9*, *CCDC18*, *ICE2*, *SERPINB7*, *RHOV*, and *CBLC* are understudied in leukemia. It was reported that the deletion of *RFX7*, encoding for a transcriptional factor, decreases natural killer (NK) cell maintenance and immunity [20]. The dysfunction of NK cells is common in human tumors, and activation of these cells has become a promising strategy to prevent relapse and induce remission when treating AML [21]. *MPHOSPH9* was reported as a susceptible locus for multiple sclerosis [22]. *CDCC18* was reported as a susceptibility gene for familial colorectal cancer. *PDS5A* is a cell cycle-related gene and precocious dissociation of PDS5A is a translocation partner of MLL in AML [23]. FANCL is a family member of DNA repair molecules and is frequently mutated in myelodysplastic syndrome (MDS), a pre-malignant hematopoietic disease; patients with MDS have increased risk of AML [24]. Moreover, hypermethylation of the *FANCL* promoter region was also suggested to be associated with sporadic acute leukemia [25]. *MUC2* belongs to the mucin family, is located on 11p15.5, and is associated with childhood AML [26]. *CEACAM5* has been widely studied in various kinds of cancers, including breast cancer [27], colorectal cancer [28, 29], pancreatic cancer [30], gastric cancer [31], and carcinoma of the tongue [32]. It is an adhesion molecule and its aberrant expression is always associated with tumor metastasis and poor prognosis. However, its function in AML has not been studied yet. In the present study, we reveal that CEACAM5 is not only one of the hub genes in AML but is also associated with unfavorable prognosis. ALDH3A1 was reported to be important in metabolizing reactive aldehydes and reactive oxygen species in hematopoietic stem cells. Its loss was associated with drug resistance and poor prognosis in AML [33].

Further, we investigated transcription factors and non-coding RNAs associated with AML. CEBPA mutations [34, 35] and GAS6-AS2 dysregulation [9] were previously reported to be strongly associated with AML prognosis and chemotherapy resistance. These results indicate that the hub genes identified in the present study are highly associated with the AML phenotype and could be used as potential therapeutic targets and biomarkers for AML.

## MATERIALS AND METHODS

### TCGA and GTEx datasets

RNA-seq data of 151 samples from adult patients with AML were downloaded from TCGA (https://portal.gdc.cancer.gov/) database. The age of the patients ranged from 18 to 88 years, and 45.0% of the patients were females. Control data from whole-blood samples of healthy subjects were obtained from GTEx (https://www.gtexportal.org/home/) database. A total of 755 control samples were included in the present study. For each sample, probe data less than 25% were excluded from the study. After annotating a probe name to each gene symbol according to the annotation files, RNA-seq data from different sources were unified by realigning raw reads, removing degraded samples, and performing batch effect correction to correct non-biological variation. Batch effect correction was performed using the SVAseq R package. AML-related genes in the NCBI GENE and OMIM databases were also downloaded for co-expression network construction.

### Identification of DEGs and co-expression network construction

The DEGs between AML and control samples were identified using the edgeR package in R Bioconductor. Genes with log fold change |logFC| > 2 and P-value < 0.05 were considered as DEGs. The co-expression network was subsequently constructed based on the DEGs using the WGCNA package. The normalized count was used when WGCNA was conducted and was standardized by the TMM method of the R package edgeR. The parameters used for WGCNA were as follows: minModuleSize = 25, mergeCutHeight = 0.25, corType = “pearson”. To identify which co-expression module had the highest relevance to the clinical phenotype, we applied the module-trait association method. Genes were clustered, and a heat-map was drawn to illustrate the association between modules and phenotype. All analyses were conducted using R Bioconductor.

### Gene ontology (GO) and Kyoto encyclopedia of genes and genomes (KEGG) enrichment analysis for co-expression modules

To get a comprehensive understanding of the function of genes associated with AML, a functional annotation was carried out for the two most relevant modules using Database for Annotation, Visualization, and Integrated Discovery (DAVID) (https://david.ncifcrf.gov/). GO and KEGG analyses were accordingly applied. Results with P-value < 0.05 were considered as significant terms and pathways, respectively.

### PIVOT analysis for identifying transcription factors, non-coding RNAs, and drugs associated with modules

To investigate the gene transcription and post-transcriptional regulations in the co-expression modules, we carried out PIVOT analysis. Non-coding RNA-gene interactions and transcription factor target data were downloaded from RAID and TRRUST databases, respectively. P-value < 0.01 indicated a significant interaction between the PIVOT regulator and the module. R Bioconductor was used for predicting the target non-coding RNAs/transcriptions factors associated with the modules. Module-related drugs were also screened by the same method. Drugs associated with AML were extracted from the DRUGBANK Database.

### Survival analysis

By considering patients with available gene expression data and clinical data, a total of 132 samples were enrolled in the survival analysis. Hub genes identified from the two most relevant modules were selected for the survival analysis. Survival data including the overall survival (OS) time and the living status were downloaded from TCGA database. Survival analysis was performed using the Kaplan-Meier method using R Bioconductor. Adjusted P values <0.05 were considered statistically significant.

## Supplementary Material

Supplementary Table 1

Supplementary Table 2

Supplementary Table 3

Supplementary Table 4
