# Supplementary material for: Identification of hub genes associated with adult acute myeloid leukemia progression through weighted gene co-expression network analysis
Source: Aging (Albany NY). 2021 Feb 11;13(4):5686–97. doi: 10.18632/aging.202493 (PMC7950274; doi:10.18632/aging.202493)
Supplement: Supplementary Table 1 [file aging-13-202493-s001.docx]

Supplementary Table 1. Detailed gene information for each module.

| ID | module |
| --- | --- |
| GATA1 | black |
| HBA1 | black |
| SELENBP1 | black |
| FIS1 | black |
| HBD | black |
| HAGH | black |
| EMC3 | black |
| TESC | black |
| HBB | black |
| FBXO7 | black |
| SHARPIN | black |
| GUK1 | black |
| ASCC2 | black |
| ADIPOR1 | black |
| CDC34 | black |
| FKBP8 | black |
| UBXN6 | black |
| DMTN | black |
| RILP | black |
| FAM210B | black |
| TENT5C | black |
| OPTN | black |
| SNCA | black |
| ELOF1 | black |
| HBA2 | black |
| STRADB | black |
| MXI1 | black |
| PCGF5 | black |
| SLC6A8 | black |
| ST6GALNAC4 | black |
| BAG1 | black |
| YBX3 | black |
| SLC25A39 | black |
| BCL2L1 | black |
| UBA52 | black |
| BABAM1 | black |
| SERF2 | black |
| GYPC | black |
| ALAS2 | black |
| FOXO4 | black |
| TMOD1 | black |
| LILRA6 | blue |
| UBTD1 | blue |
| ZNF467 | blue |
| AP5B1 | blue |
| C5AR2 | blue |
| BCAP31 | blue |
| PPP1R3B | blue |
| OAZ2 | blue |
| NEDD9 | blue |
| HLX | blue |
| GLT1D1 | blue |
| SNX11 | blue |
| SIGLEC9 | blue |
| LMAN2 | blue |
| RGS19 | blue |
| CXCR2 | blue |
| RIN3 | blue |
| GLIPR2 | blue |
| NOSIP | blue |
| TLR1 | blue |
| EXT1 | blue |
| TLR6 | blue |
| HAUS4 | blue |
| B3GALT4 | blue |
| BATF | blue |
| IFNGR1 | blue |
| VSIR | blue |
| COPE | blue |
| MED11 | blue |
| ARHGAP24 | blue |
| TNFSF14 | blue |
| CD63 | blue |
| ORMDL2 | blue |
| PLIN5 | blue |
| MXD3 | blue |
| KCNH7 | blue |
| SDCBP | blue |
| COTL1 | blue |
| CCNDBP1 | blue |
| G6PD | blue |
| HTATIP2 | blue |
| FPR1 | blue |
| FGD3 | blue |
| N4BP1 | blue |
| EVI2B | blue |
| USB1 | blue |
| PTAFR | blue |
| SORT1 | blue |
| CFP | blue |
| TADA3 | blue |
| GPAT3 | blue |
| MKNK1 | blue |
| TMEM88 | blue |
| H3F3A | blue |
| MAPK3 | blue |
| YIPF1 | blue |
| ZNF564 | blue |
| ITGAM | blue |
| WAS | blue |
| ANXA3 | blue |
| CAMKK2 | blue |
| POLD4 | blue |
| NR1H2 | blue |
| TUBA4A | blue |
| HSPA1L | blue |
| LRCH4 | blue |
| RNF13 | blue |
| IL16 | blue |
| RAB5C | blue |
| ADAP1 | blue |
| ADGRE5 | blue |
| ARF5 | blue |
| FBXW5 | blue |
| MYO1F | blue |
| JDP2 | blue |
| ZBTB7B | blue |
| SLC25A44 | blue |
| NDEL1 | blue |
| STX11 | blue |
| MBD6 | blue |
| DBNL | blue |
| CLIC1 | blue |
| BASP1 | blue |
| MYL12A | blue |
| SEMA4A | blue |
| SQOR | blue |
| SDF2 | blue |
| FBXL5 | blue |
| CYBA | blue |
| TP53I11 | blue |
| MSL1 | blue |
| HCK | blue |
| PTPN6 | blue |
| RAB43 | blue |
| TM9SF1 | blue |
| MYD88 | blue |
| LRG1 | blue |
| TSC22D4 | blue |
| ARHGDIB | blue |
| CCNJL | blue |
| PLBD1 | blue |
| FLOT2 | blue |
| ARHGAP9 | blue |
| FRAT2 | blue |
| TBXAS1 | blue |
| ATP6V0E1 | blue |
| VPS9D1 | blue |
| S100A4 | blue |
| BLOC1S1 | blue |
| IL1R2 | blue |
| DTX2 | blue |
| MDP1 | blue |
| MTHFS | blue |
| IGFLR1 | blue |
| RPS6KA1 | blue |
| PRKCD | blue |
| FGR | blue |
| ITPKC | blue |
| SIRPA | blue |
| S100A11 | blue |
| PSMB3 | blue |
| DGAT2 | blue |
| JAK3 | blue |
| MIA2 | blue |
| ZDHHC18 | blue |
| PPP4C | blue |
| OPRL1 | blue |
| RNF181 | blue |
| LAMTOR4 | blue |
| CSF3R | blue |
| ALPK1 | blue |
| ALOX5AP | blue |
| LRFN1 | blue |
| XKR8 | blue |
| SULT1B1 | blue |
| ADAM8 | blue |
| IFITM1 | blue |
| CREB5 | blue |
| APBB1IP | blue |
| STK16 | blue |
| VASP | blue |
| SLC9A8 | blue |
| UPP1 | blue |
| TNFRSF1A | blue |
| KIAA0040 | blue |
| SRA1 | blue |
| TRIM25 | blue |
| GALK1 | blue |
| MAP3K3 | blue |
| EXTL3 | blue |
| SIGLEC5 | blue |
| NUP214 | blue |
| NSUN7 | blue |
| TGFA | blue |
| RAC2 | blue |
| NCF4 | blue |
| C1orf162 | blue |
| MANSC1 | blue |
| TREML2 | blue |
| OGFR | blue |
| IL13RA1 | blue |
| GRINA | blue |
| PGS1 | blue |
| RNASET2 | blue |
| TFEB | blue |
| CSF2RA | blue |
| PPP1R3D | blue |
| CTSZ | blue |
| NLRC4 | blue |
| LILRA2 | blue |
| SH3BGRL3 | blue |
| TNFRSF10C | blue |
| STK40 | blue |
| ACTB | blue |
| MEF2B | blue |
| STAT5B | blue |
| MMP25 | blue |
| ECE1 | blue |
| MIIP | blue |
| INKA2 | blue |
| IMPA2 | blue |
| PRELID1 | blue |
| FRAT1 | blue |
| RASGRP4 | blue |
| IKBKG | blue |
| VAV1 | blue |
| LAPTM5 | blue |
| PFN1 | blue |
| BRMS1 | blue |
| DYNLT1 | blue |
| PSTPIP1 | blue |
| RABAC1 | blue |
| MLF2 | blue |
| APMAP | blue |
| LILRB3 | blue |
| ARSG | blue |
| ARPC5 | blue |
| P2RY13 | blue |
| ARL8A | blue |
| CCDC71L | blue |
| STX3 | blue |
| MBOAT7 | blue |
| NLRP6 | blue |
| FPR2 | blue |
| GMFG | blue |
| AGTRAP | blue |
| AQP9 | blue |
| C19orf38 | blue |
| ROM1 | blue |
| B3GNT8 | blue |
| CASP1 | blue |
| UBE2R2 | blue |
| FURIN | blue |
| S100A6 | blue |
| PRKACA | blue |
| SHISA5 | blue |
| PISD | blue |
| MAP11 | blue |
| APOBEC3A | blue |
| GCA | blue |
| S1PR4 | blue |
| CFLAR | blue |
| AGPAT2 | blue |
| KCNJ2 | blue |
| CA4 | blue |
| ARAP1 | blue |
| ABTB1 | blue |
| SELPLG | blue |
| SRXN1 | blue |
| RALB | blue |
| LIMD2 | blue |
| PGD | blue |
| ADGRG3 | blue |
| PXN | blue |
| CNN2 | blue |
| NCF1 | blue |
| KCNJ15 | blue |
| NHSL2 | blue |
| LIN7A | blue |
| GMIP | blue |
| FLOT1 | blue |
| STX10 | blue |
| GRAMD1A | blue |
| WWC3 | blue |
| MEGF9 | blue |
| TCIRG1 | blue |
| TIMM17B | blue |
| KCNQ1 | blue |
| TNFSF10 | blue |
| IGSF6 | blue |
| ARMH2 | blue |
| RNPEPL1 | blue |
| TMEM154 | blue |
| PGK1 | blue |
| ACOX1 | blue |
| SH3GLB1 | blue |
| ARRB2 | blue |
| S100A9 | blue |
| GSK3A | blue |
| PYCARD | blue |
| RARA | blue |
| THEMIS2 | blue |
| SH3BP5L | blue |
| CCND3 | blue |
| RGS2 | blue |
| CMTM2 | blue |
| ACAA1 | blue |
| ARHGAP27 | blue |
| IMPDH1 | blue |
| ZNF524 | blue |
| CARD6 | blue |
| TMEM91 | blue |
| TMEM120A | blue |
| EFHD2 | blue |
| PPP1R18 | blue |
| PRR13 | blue |
| GABARAP | blue |
| ATP6V0B | blue |
| CD53 | blue |
| HRH2 | blue |
| CEBPD | blue |
| MSRB1 | blue |
| ST6GALNAC2 | blue |
| NFE2 | blue |
| DHRS13 | blue |
| LIN37 | blue |
| CEP19 | blue |
| PLXNC1 | blue |
| GNAI2 | blue |
| EMC6 | blue |
| LILRA5 | blue |
| XPO6 | blue |
| RBM4 | blue |
| RAB31 | blue |
| FCER1G | blue |
| POLR2J | blue |
| SLC12A9 | blue |
| MICAL1 | blue |
| ATP6V0D1 | blue |
| FERMT3 | blue |
| TRIOBP | blue |
| C1RL | blue |
| TPK1 | blue |
| SIRPB1 | blue |
| RAB4B | blue |
| ITM2B | blue |
| ILK | blue |
| SERPINA1 | blue |
| NRBF2 | blue |
| JAML | blue |
| CRISPLD2 | blue |
| MX2 | blue |
| SASH3 | blue |
| NIBAN1 | blue |
| SLC22A4 | blue |
| BIN2 | blue |
| CYBC1 | blue |
| LTB4R | blue |
| RGS14 | blue |
| NPL | blue |
| RNF167 | blue |
| SLA | blue |
| RASGEF1A | blue |
| PICALM | blue |
| CPPED1 | blue |
| OSTF1 | blue |
| CHIC2 | blue |
| ANXA11 | blue |
| FADD | blue |
| NLRP12 | blue |
| PFKFB4 | blue |
| SLC6A6 | blue |
| FCGR2A | blue |
| TLR5 | blue |
| SHKBP1 | blue |
| HLA-C | blue |
| SLC19A1 | blue |
| LRRC25 | blue |
| ARPC1B | blue |
| NSFL1C | blue |
| CSK | blue |
| CEACAM4 | blue |
| KIAA2013 | blue |
| ARPC3 | blue |
| GPSM3 | blue |
| DEF6 | blue |
| TECPR2 | blue |
| ITGAX | blue |
| CHMP2A | blue |
| TYROBP | blue |
| LSP1 | blue |
| PHF23 | blue |
| CSF2RB | blue |
| ADAM19 | blue |
| CORO1A | blue |
| TMEM8A | blue |
| GPR108 | blue |
| CPQ | blue |
| KCTD21 | blue |
| JPT1 | blue |
| NOL12 | blue |
| ISY1 | blue |
| CAMKK1 | blue |
| FKBP5 | blue |
| RTF2 | blue |
| PEAK3 | blue |
| IFNGR2 | blue |
| FCGRT | blue |
| SMAP2 | blue |
| ATP6V1B2 | blue |
| MVP | blue |
| 1-Mar | blue |
| CTDSP1 | blue |
| ITGB2 | blue |
| IRF2 | blue |
| NFAM1 | blue |
| TBKBP1 | blue |
| TFE3 | blue |
| FBXL15 | blue |
| ARPC4 | blue |
| TWF2 | blue |
| HK3 | blue |
| PPM1M | blue |
| PLB1 | blue |
| TPD52L2 | blue |
| RASSF3 | blue |
| MRPL28 | blue |
| TKT | blue |
| SCAND1 | blue |
| TRIM21 | blue |
| ZNF787 | blue |
| PREX1 | blue |
| EHD1 | blue |
| DEF8 | blue |
| TPST1 | blue |
| RAB24 | blue |
| CLEC4E | blue |
| PLPPR2 | blue |
| POR | blue |
| RAB5IF | blue |
| RHOG | blue |
| PSENEN | blue |
| MSL3 | blue |
| AURKAIP1 | blue |
| CKLF | blue |
| CYTH4 | blue |
| PHC2 | blue |
| CYB5R1 | blue |
| CANT1 | blue |
| IL10RB | blue |
| IL4R | blue |
| LRPAP1 | blue |
| RNASEK | blue |
| ICAM3 | blue |
| MRVI1 | blue |
| KREMEN1 | blue |
| PADI2 | blue |
| ZYX | blue |
| DNTTIP1 | blue |
| MCEMP1 | blue |
| GNG5 | blue |
| HAL | blue |
| NUDT16 | blue |
| LRRK2 | blue |
| SERTAD1 | blue |
| IFITM2 | blue |
| PTPRJ | blue |
| MGAM | blue |
| KIAA0513 | blue |
| QPCT | blue |
| TSEN34 | blue |
| TRAPPC1 | blue |
| PRCP | blue |
| BORCS6 | blue |
| RAB11FIP4 | blue |
| NBEAL2 | blue |
| NADK | blue |
| SLC44A2 | blue |
| EIF4EBP3 | blue |
| UBE2D1 | blue |
| NCF2 | blue |
| STEAP4 | blue |
| NPFF | blue |
| LCP1 | blue |
| WDFY3 | blue |
| PLEKHO2 | blue |
| PANX2 | blue |
| STXBP2 | blue |
| LRRC4 | blue |
| TNFSF13 | blue |
| CASP4 | blue |
| PYGL | blue |
| SLC16A3 | blue |
| ALOX5 | blue |
| SPI1 | blue |
| ZFPL1 | blue |
| RAB7A | blue |
| LIMK2 | blue |
| TLR8 | blue |
| TYMP | blue |
| GRK6 | blue |
| ORAI2 | blue |
| CST7 | blue |
| ITPK1 | blue |
| BRI3 | blue |
| ROPN1L | blue |
| ARHGAP25 | blue |
| TMED1 | blue |
| SLC26A8 | blue |
| SDHAF2 | blue |
| TUBA1A | blue |
| TAGLN2 | blue |
| MNDA | blue |
| REPS2 | blue |
| RTN3 | blue |
| SLC15A3 | blue |
| TM6SF1 | blue |
| SBNO2 | blue |
| HLA-E | blue |
| CDA | blue |
| IL18RAP | blue |
| FAM49A | blue |
| TRIM34 | blue |
| ABHD16A | blue |
| DHRS7 | blue |
| PLP2 | blue |
| DNAH17 | blue |
| FAM49B | blue |
| CHMP1A | blue |
| KLHL21 | blue |
| CFL1 | blue |
| OSCAR | blue |
| VNN2 | blue |
| CAPZB | blue |
| TALDO1 | blue |
| ALPL | blue |
| LITAF | blue |
| AATK | blue |
| LRP10 | blue |
| TSPO | blue |
| CCDC69 | blue |
| MYL12B | blue |
| SELL | blue |
| ARHGAP30 | blue |
| DOK3 | blue |
| GNB2 | blue |
| NAA60 | blue |
| NUAK2 | blue |
| FUT7 | blue |
| LYN | blue |
| PDLIM7 | blue |
| KCNB1 | blue |
| NQO2 | blue |
| UBXN2B | blue |
| RNF149 | blue |
| SECTM1 | blue |
| SEMA4B | blue |
| IRF2BPL | blue |
| DNAJC5 | blue |
| DYSF | blue |
| PSMD4 | blue |
| ZNF438 | blue |
| PAGR1 | blue |
| CAP1 | blue |
| VMP1 | blue |
| TADA2B | blue |
| CXCR1 | blue |
| HCLS1 | blue |
| CDC42SE1 | blue |
| TLR4 | blue |
| EGLN2 | blue |
| AP3S2 | blue |
| NPEPL1 | blue |
| ERGIC1 | blue |
| RASSF2 | blue |
| RIPK3 | blue |
| APOBR | blue |
| CAPG | blue |
| MOB3A | blue |
| TPRG1L | blue |
| TNFAIP8L2 | blue |
| CLDN9 | blue |
| ACSL1 | blue |
| UBE2B | blue |
| GK | blue |
| TMEM35B | blue |
| PACSIN2 | blue |
| SRPK1 | blue |
| NAMPT | blue |
| ECHDC3 | blue |
| SIPA1L2 | blue |
| ST6GALNAC3 | blue |
| GNG10 | blue |
| JOSD2 | blue |
| IL18R1 | blue |
| ARHGAP26 | blue |
| TMEM71 | blue |
| KLHL2 | blue |
| SLC37A3 | blue |
| TAS2R40 | blue |
| ASAH1 | blue |
| CCPG1 | blue |
| DDAH2 | blue |
| CR1 | blue |
| BCL6 | blue |
| GALNT4 | blue |
| CD55 | blue |
| PTPRC | blue |
| TNS4 | brown |
| ALDH3A1 | brown |
| RAB25 | brown |
| NKX2-1 | brown |
| LY6D | brown |
| ANKRD45 | brown |
| RNF183 | brown |
| CATSPERD | brown |
| CYP1A1 | brown |
| SDR16C5 | brown |
| LAD1 | brown |
| LGALS7B | brown |
| KLK13 | brown |
| GRAMD2A | brown |
| LGALS7 | brown |
| WFDC2 | brown |
| CFAP73 | brown |
| FAM3D | brown |
| CLCA4 | brown |
| IL20RA | brown |
| SERPINB4 | brown |
| CXCL6 | brown |
| FOXA1 | brown |
| C9orf135 | brown |
| KLK10 | brown |
| FAM81B | brown |
| CFAP52 | brown |
| PLAAT2 | brown |
| SERPINB5 | brown |
| MLPH | brown |
| FGFBP1 | brown |
| TJP3 | brown |
| SFTA3 | brown |
| EFCAB6 | brown |
| CBLC | brown |
| CDHR4 | brown |
| CHP2 | brown |
| SERPINB3 | brown |
| MYH14 | brown |
| DMBT1 | brown |
| SPRR1B | brown |
| DEFB4A | brown |
| C11orf16 | brown |
| PIGR | brown |
| AGR3 | brown |
| FUT2 | brown |
| SERPINB7 | brown |
| TSPAN8 | brown |
| GABRP | brown |
| GRB7 | brown |
| MUC5AC | brown |
| IL6 | brown |
| SCGB1A1 | brown |
| KRT7 | brown |
| AQP5 | brown |
| SFTPA1 | brown |
| MAL2 | brown |
| S100A7 | brown |
| SLC5A1 | brown |
| EHF | brown |
| C20orf85 | brown |
| TMC5 | brown |
| DAW1 | brown |
| SERPINB13 | brown |
| MS4A8 | brown |
| SLC34A2 | brown |
| MUC2 | brown |
| TRIM29 | brown |
| BPIFA1 | brown |
| CKMT1B | brown |
| MSMB | brown |
| PRB4 | brown |
| PRB1 | brown |
| PRSS22 | brown |
| KRT15 | brown |
| LAMC2 | brown |
| MMP13 | brown |
| SCGB3A1 | brown |
| DNAH12 | brown |
| MMP1 | brown |
| CXCL1 | brown |
| ADH7 | brown |
| SPRR2F | brown |
| PERP | brown |
| C11orf97 | brown |
| KRT19 | brown |
| KRT5 | brown |
| CEACAM5 | brown |
| DRC1 | brown |
| TMEM232 | brown |
| RHOV | brown |
| SFN | brown |
| MMP10 | brown |
| TEKT2 | brown |
| CCDC60 | brown |
| WDR78 | brown |
| TTLL10 | brown |
| CX3CL1 | brown |
| TFAP2C | brown |
| CSF3 | brown |
| SNTN | brown |
| CFAP300 | brown |
| S100A2 | brown |
| S100A14 | brown |
| ERBB3 | brown |
| MUCL1 | brown |
| CCL20 | brown |
| CAPN8 | brown |
| MLF1 | brown |
| CFAP157 | brown |
| ADGRF1 | brown |
| GDF15 | brown |
| CLDN4 | brown |
| CCDC113 | brown |
| SOX2 | brown |
| KLK11 | brown |
| FUT6 | brown |
| CDC42EP5 | brown |
| TMPRSS2 | brown |
| C5orf49 | brown |
| SFTPA2 | brown |
| DNAAF3 | brown |
| TSPAN1 | brown |
| KRT16 | brown |
| LRRC71 | brown |
| BPIFB2 | brown |
| RSPH4A | brown |
| TUBA4B | brown |
| TNFRSF6B | brown |
| SPDEF | brown |
| SPATA18 | brown |
| BPIFB1 | brown |
| SLC6A14 | brown |
| RIPK4 | brown |
| PDLIM3 | green |
| MYLPF | green |
| XIRP1 | green |
| MYL1 | green |
| C10orf71 | green |
| EEF1A2 | green |
| MYBPC1 | green |
| MYBPC2 | green |
| HRC | green |
| TNNT1 | green |
| POPDC3 | green |
| SRL | green |
| HSPB3 | green |
| TNNI2 | green |
| MYH2 | green |
| IGFN1 | green |
| MYH1 | green |
| CASQ1 | green |
| ACTN2 | green |
| TRIM54 | green |
| AGBL1 | green |
| UNC45B | green |
| SMTNL2 | green |
| TNNC2 | green |
| HSPB8 | green |
| MYL3 | green |
| TNNC1 | green |
| FBP2 | green |
| HSPB6 | green |
| CKM | green |
| SMYD1 | green |
| SGCA | green |
| PYGM | green |
| RPL3L | green |
| CRYAB | green |
| DUSP26 | green |
| NRAP | green |
| TCAP | green |
| ASB10 | green |
| SYPL2 | green |
| CACNA1S | green |
| MYOM3 | green |
| SMPX | green |
| MYH7 | green |
| MYOD1 | green |
| MYPN | green |
| TMOD4 | green |
| LDB3 | green |
| KLHL40 | green |
| HHATL | green |
| MYOZ1 | green |
| HSPB7 | green |
| RXRG | green |
| VGLL2 | green |
| PERM1 | green |
| MB | green |
| JPH2 | green |
| DHRS7C | green |
| SLN | green |
| ACTA1 | green |
| MYL2 | green |
| TNNI1 | green |
| COX6A2 | green |
| FLNC | green |
| DES | green |
| PEBP4 | green |
| RIPOR2 | grey |
| TLR2 | grey |
| OSBPL2 | grey |
| ACTN1 | grey |
| ZFP36 | grey |
| TAX1BP3 | grey |
| GABRA4 | grey |
| VPREB3 | grey |
| MEF2C | grey |
| MT3 | grey |
| DNMT3B | grey |
| ZFP36L1 | grey |
| HOXA2 | grey |
| TDP2 | grey |
| USP50 | grey |
| CD68 | grey |
| FAT3 | grey |
| TMEM169 | grey |
| CAPN11 | grey |
| CYP27B1 | grey |
| SPEF2 | grey |
| FDX2 | grey |
| ANKRD1 | grey |
| GABRE | grey |
| PRAME | grey |
| CRISP3 | grey |
| GABRA2 | grey |
| LIF | grey |
| VAT1L | grey |
| ADAMTS14 | grey |
| PILRA | grey |
| GSDMC | grey |
| CRTAC1 | grey |
| KIF26B | grey |
| OR13A1 | grey |
| DUSP27 | grey |
| MAP1A | grey |
| ACTN3 | grey |
| SPINK2 | grey |
| CTRB1 | grey |
| ATP1B4 | grey |
| BEST1 | grey |
| PRKG2 | grey |
| PCDHGC3 | grey |
| TEX15 | grey |
| FOSB | grey |
| ACR | grey |
| MYOCD | grey |
| NUDT7 | grey |
| FMO5 | grey |
| STMN4 | grey |
| MEI4 | grey |
| CERS1 | grey |
| IRF6 | grey |
| AGRN | grey |
| MKRN3 | grey |
| MEFV | grey |
| PRDM1 | grey |
| KCNE1B | grey |
| FAM151A | grey |
| ABO | grey |
| PHGR1 | grey |
| MS4A6E | grey |
| NDST2 | grey |
| TNFRSF8 | grey |
| FOLR3 | grey |
| IFI27 | grey |
| NKAIN1 | grey |
| ANKRD9 | grey |
| ENAM | grey |
| KCNJ1 | grey |
| LMLN | grey |
| EDA2R | grey |
| IGSF1 | grey |
| VSTM2B | grey |
| PRL | grey |
| IGLL1 | grey |
| OR9A4 | grey |
| SELENOP | grey |
| ST6GAL2 | grey |
| PHYHIPL | grey |
| DOC2A | grey |
| SMR3B | grey |
| SLC38A5 | grey |
| GLOD5 | grey |
| HAS2 | grey |
| METRNL | grey |
| KLC3 | grey |
| FOXF2 | grey |
| LHFPL4 | grey |
| NPM1 | grey |
| DPT | grey |
| NKG7 | grey |
| LHX6 | grey |
| LPP | grey |
| SGSH | grey |
| EPYC | grey |
| DNAH7 | grey |
| TMEM59L | grey |
| DAPK2 | grey |
| PHYHIP | grey |
| C1QC | grey |
| GGT1 | grey |
| MSH4 | grey |
| C3 | grey |
| PHF24 | grey |
| MYO18B | grey |
| IL24 | grey |
| F11R | grey |
| RPH3A | grey |
| CD22 | grey |
| USP6 | grey |
| PSME1 | grey |
| MROH9 | grey |
| CCDC192 | grey |
| HTR1F | grey |
| PPP1R1A | grey |
| PI3 | grey |
| ADM | grey |
| TMPRSS11D | grey |
| S100A12 | grey |
| PTX4 | grey |
| ZNF404 | grey |
| ABCC3 | grey |
| CSNK1A1L | grey |
| HMGN5 | grey |
| MISP3 | grey |
| C9orf43 | grey |
| SLPI | grey |
| TLE2 | grey |
| DEFA1 | grey |
| CYP4F2 | grey |
| BICDL1 | grey |
| HEY2 | grey |
| ZIC1 | grey |
| ADGRG6 | grey |
| SLC9C2 | grey |
| PRELID3A | grey |
| MME | grey |
| NEU4 | grey |
| C6orf141 | grey |
| TEN1 | grey |
| OXER1 | grey |
| INHBC | grey |
| TP53TG5 | grey |
| KCNJ6 | grey |
| PROK2 | grey |
| INTU | grey |
| PCDHB14 | grey |
| KCNK16 | grey |
| ZNF695 | grey |
| NRIP1 | grey |
| ZDHHC19 | grey |
| SPATA2L | grey |
| ADORA2A | grey |
| CRHBP | grey |
| GPR137C | grey |
| TMEM221 | grey |
| CYP4Z1 | grey |
| MAB21L1 | grey |
| P2RX2 | grey |
| WFIKKN2 | grey |
| NPAS1 | grey |
| ACTG2 | grey |
| PCDHGB4 | grey |
| FCGR2B | grey |
| ARNTL2 | grey |
| MIA | grey |
| SNCB | grey |
| PCDHB9 | grey |
| STX1A | grey |
| MAL | grey |
| MPP4 | grey |
| WDR17 | grey |
| TAP1 | grey |
| DMXL2 | grey |
| MAP1LC3A | grey |
| FFAR2 | grey |
| MEIS1 | grey |
| ADGRB2 | grey |
| ASAP2 | grey |
| CPLX1 | grey |
| OR1Q1 | grey |
| CRPPA | grey |
| LRRC63 | grey |
| UPK3BL1 | grey |
| NDST3 | grey |
| MT1B | grey |
| FNDC4 | grey |
| CNN1 | grey |
| NPHS2 | grey |
| DSC3 | grey |
| CA7 | grey |
| BSCL2 | grey |
| TMEFF2 | grey |
| GRIK5 | grey |
| PRDM5 | grey |
| NXPE1 | grey |
| KDELR3 | grey |
| GJB6 | grey |
| CKAP4 | grey |
| ETV6 | grey |
| TTC23L | grey |
| GPX3 | grey |
| HIST2H2BE | grey |
| MAGEC2 | grey |
| SLC34A3 | grey |
| CRHR2 | grey |
| MYZAP | grey |
| KRTAP5-7 | grey |
| C19orf81 | grey |
| NLGN4X | grey |
| AJAP1 | grey |
| NPTX2 | grey |
| RIMKLB | grey |
| HSPA4L | grey |
| ADGRD2 | grey |
| C7orf25 | grey |
| DOK2 | grey |
| WIPI1 | grey |
| NAV1 | grey |
| NID2 | grey |
| GRAP2 | grey |
| CXCL16 | grey |
| GPRC5A | grey |
| DMC1 | grey |
| NEURL2 | grey |
| SIPA1 | grey |
| SCN9A | grey |
| RAB23 | grey |
| PRB3 | grey |
| NXPE4 | grey |
| PLAAT4 | grey |
| MAP2K2 | grey |
| CRTAM | grey |
| TSPAN11 | grey |
| ZNF781 | grey |
| TUBA3E | grey |
| IFI30 | grey |
| PIM1 | grey |
| ZNF474 | grey |
| TDRD1 | grey |
| LAT | grey |
| GFRA2 | grey |
| DNAJB2 | grey |
| ITK | grey |
| PIK3R2 | grey |
| LTBP1 | grey |
| TUBAL3 | grey |
| ROBO2 | grey |
| FAM180B | grey |
| KITLG | grey |
| MINDY4B | grey |
| NGF | grey |
| LMOD1 | grey |
| MT1X | grey |
| RELB | grey |
| CTSD | grey |
| CDC42EP3 | grey |
| MAB21L3 | grey |
| LRRC9 | grey |
| PIRT | grey |
| HOXB6 | grey |
| C3AR1 | grey |
| ELOB | grey |
| RBPMS | grey |
| NAPB | grey |
| NDUFA7 | grey |
| PTCH2 | grey |
| REN | grey |
| TNNI3 | grey |
| DYNC2H1 | grey |
| EDN1 | grey |
| METTL24 | grey |
| MGAM2 | grey |
| NUAK1 | grey |
| C1orf122 | grey |
| ZNF727 | grey |
| HOXB3 | grey |
| ODF3L1 | grey |
| CCL5 | grey |
| ZNF98 | grey |
| PRIMA1 | grey |
| NLRP3 | grey |
| WEE2 | grey |
| SYCP2L | grey |
| PRR9 | grey |
| PLA2G1B | grey |
| SCEL | grey |
| PLSCR1 | grey |
| LRTM2 | grey |
| SLC27A2 | grey |
| HES1 | grey |
| TBC1D2 | grey |
| GRIN2C | grey |
| GLI2 | grey |
| LCE1C | grey |
| LGALS9B | grey |
| SCN3A | grey |
| ANKRD55 | grey |
| STAR | grey |
| NKX3-2 | grey |
| LURAP1 | grey |
| PDCD1 | grey |
| PGGHG | grey |
| MMP2 | grey |
| RASL11A | grey |
| ARHGAP8 | grey |
| FBXO39 | grey |
| CAPN13 | grey |
| ASB9 | grey |
| ISG15 | grey |
| SGCD | grey |
| CLPS | grey |
| CCR1 | grey |
| PDZD9 | grey |
| NAIP | grey |
| RASL11B | grey |
| SLC4A1 | grey |
| INSL5 | grey |
| SLC35F2 | grey |
| ALOX15B | grey |
| CLTB | grey |
| LIPM | grey |
| TANC1 | grey |
| DST | grey |
| CSDC2 | grey |
| OLFML2A | grey |
| EXPH5 | grey |
| SOSTDC1 | grey |
| TPH1 | grey |
| VGLL3 | grey |
| FREM1 | grey |
| TWIST2 | grey |
| FAM50A | grey |
| PTMS | grey |
| FKBP1B | grey |
| SLC22A14 | grey |
| PCDHGA6 | grey |
| KIAA0408 | grey |
| DTNA | grey |
| TMSB4X | grey |
| ITIH4 | grey |
| UNCX | grey |
| ANGPT2 | grey |
| TLR9 | grey |
| ZNF503 | grey |
| MKRN1 | grey |
| IL5 | grey |
| C16orf71 | grey |
| JUP | grey |
| BSND | grey |
| SSX1 | grey |
| BICDL2 | grey |
| DEDD2 | grey |
| BPGM | grey |
| DEFA4 | grey |
| OR2D2 | grey |
| PCDH7 | grey |
| PTPRS | grey |
| PCDHGB2 | grey |
| IDO1 | grey |
| OR5B21 | grey |
| CYP3A5 | grey |
| TXNDC5 | grey |
| KCNMB2 | grey |
| BIVM | grey |
| MS4A4E | grey |
| PGM5 | grey |
| GPR88 | grey |
| RPRM | grey |
| STAT3 | grey |
| ITGA2B | grey |
| TG | grey |
| CLU | grey |
| JCAD | grey |
| MASP2 | grey |
| OSM | grey |
| H3F3B | grey |
| SVOPL | grey |
| CSRP3 | grey |
| CATSPER1 | grey |
| MOK | grey |
| STUM | grey |
| FLT1 | grey |
| DPPA4 | grey |
| POLE | grey |
| GLYATL1B | grey |
| ARHGEF1 | grey |
| TNFRSF12A | grey |
| FCMR | grey |
| DAAM1 | grey |
| KRT85 | grey |
| CD8A | grey |
| TMTC1 | grey |
| PLGLB2 | grey |
| PDZD7 | grey |
| KLRF2 | grey |
| ZNF391 | grey |
| SCN1B | grey |
| ACHE | grey |
| FER1L6 | grey |
| AMY2A | grey |
| ATL1 | grey |
| TTC23 | grey |
| OPN1SW | grey |
| CYTIP | grey |
| FAM170B | grey |
| KDM6B | grey |
| DMRTC2 | grey |
| CCL2 | grey |
| GPR32 | grey |
| CBFB | grey |
| SNAI3 | grey |
| DLK1 | grey |
| WBP1 | grey |
| PAXX | grey |
| GJB2 | grey |
| CP | grey |
| PRR5 | grey |
| KCNH4 | grey |
| MEOX1 | grey |
| BHLHE23 | grey |
| MARCO | grey |
| PDE7B | grey |
| MAPK13 | grey |
| PTGER1 | grey |
| SIGLEC14 | grey |
| OR51V1 | grey |
| FAM171B | grey |
| ODF3 | grey |
| GPR151 | grey |
| MYO16 | grey |
| SIRPG | grey |
| CARD16 | grey |
| B3GALT2 | grey |
| CASQ2 | grey |
| HIST2H2BF | grey |
| DGKK | grey |
| CHPT1 | grey |
| GAD1 | grey |
| CXCL12 | grey |
| GREM2 | grey |
| GABRQ | grey |
| GLRB | grey |
| ZDHHC15 | grey |
| GTF2H4 | grey |
| RNF224 | grey |
| ZNF257 | grey |
| RHBDD2 | grey |
| PRODH | grey |
| GPC6 | grey |
| STON1-GTF2A1L | grey |
| OR2AK2 | grey |
| CCL19 | grey |
| HMX3 | grey |
| HIST1H2BB | grey |
| WDR64 | grey |
| TAGLN3 | grey |
| CLEC11A | grey |
| DPP10 | grey |
| ONECUT2 | grey |
| OR51I2 | grey |
| ANKK1 | grey |
| STARD9 | grey |
| SPSB4 | grey |
| PRSS57 | grey |
| THRSP | grey |
| PLAUR | grey |
| THSD7A | grey |
| ALS2 | grey |
| PYDC1 | grey |
| SPARCL1 | grey |
| AHSP | grey |
| KLRC4 | grey |
| CCDC62 | grey |
| APBA2 | grey |
| GP9 | grey |
| ISG20 | grey |
| SIGLEC15 | grey |
| TMEM119 | grey |
| MRPL55 | grey |
| RPS28 | grey |
| OLFM1 | grey |
| CIB3 | grey |
| GSX1 | grey |
| AOC3 | grey |
| CCDC175 | grey |
| HEPACAM2 | grey |
| NLGN1 | grey |
| GOLGA8S | grey |
| TNFAIP6 | grey |
| PSMD9 | grey |
| NPAS3 | grey |
| SULT1E1 | grey |
| ECSCR | grey |
| SYNM | grey |
| ABCA9 | grey |
| SPNS3 | grey |
| SPATA16 | grey |
| PPARGC1A | grey |
| PNMA5 | grey |
| SLC44A1 | grey |
| ETV4 | grey |
| PIP | grey |
| IRX3 | grey |
| RAB33A | grey |
| MYL7 | grey |
| MTSS2 | grey |
| MN1 | grey |
| GPER1 | grey |
| KIRREL3 | grey |
| CDX2 | grey |
| B4GALNT3 | grey |
| PRR16 | grey |
| EGR1 | grey |
| PLD4 | grey |
| CYP11B1 | grey |
| RPS24 | grey |
| NXNL1 | grey |
| SCGB1C1 | grey |
| SERTAD3 | grey |
| TMEM74 | grey |
| MMP3 | grey |
| EZHIP | grey |
| ANKRD13B | grey |
| BCL2L10 | grey |
| MMP7 | grey |
| LYPD8 | grey |
| GPR161 | grey |
| DAAM2 | grey |
| KLHL41 | grey |
| NINJ2 | grey |
| PTPN20 | grey |
| NSD1 | grey |
| B3GAT1 | grey |
| PALM | grey |
| FCGR3A | grey |
| ALDOC | grey |
| AXIN2 | grey |
| NDUFA11 | grey |
| AMER2 | grey |
| DLX1 | grey |
| TMEM86B | grey |
| TRHR | grey |
| TUBB1 | grey |
| COL25A1 | grey |
| MFAP4 | grey |
| DPY19L2 | grey |
| BATF2 | grey |
| GCKR | grey |
| PYCR1 | grey |
| SLC35G2 | grey |
| TTYH1 | grey |
| TGM2 | grey |
| TFF3 | grey |
| PLXDC1 | grey |
| ADRB1 | grey |
| SRGAP1 | grey |
| SYCE2 | grey |
| PMP2 | grey |
| TTC28 | grey |
| CCDC173 | grey |
| CEP295NL | grey |
| PPP1R3C | grey |
| CHIT1 | grey |
| GTSF1 | grey |
| DPP6 | grey |
| CSPG4 | grey |
| REG1A | grey |
| TMEFF1 | grey |
| CCL1 | grey |
| IQCK | grey |
| C8orf48 | grey |
| SLCO1A2 | grey |
| PRELP | grey |
| OMD | grey |
| HIST1H4H | grey |
| VPS28 | grey |
| IYD | grey |
| KCNRG | grey |
| YPEL4 | grey |
| KCNK17 | grey |
| AFF3 | grey |
| RCVRN | grey |
| UGGT2 | grey |
| HPCAL1 | grey |
| RTN4R | grey |
| ZNF827 | grey |
| TFPI2 | grey |
| HBE1 | grey |
| KLK3 | grey |
| EMX1 | grey |
| ENPEP | grey |
| OR13G1 | grey |
| MAP7 | grey |
| ATG9A | grey |
| NKD2 | grey |
| TRIM40 | grey |
| SNN | grey |
| FOXR1 | grey |
| ACTL6B | grey |
| C16orf96 | grey |
| ZNF667 | grey |
| C15orf48 | grey |
| ENAH | grey |
| TMEM252 | grey |
| PLS3 | grey |
| PI15 | grey |
| CLEC4F | grey |
| ITLN2 | grey |
| ELAVL4 | grey |
| GPR4 | grey |
| GTPBP1 | grey |
| TAF1L | grey |
| FAM3B | grey |
| VPREB1 | grey |
| LIME1 | grey |
| MDK | grey |
| ARTN | grey |
| CCDC107 | grey |
| SOX17 | grey |
| KRT72 | grey |
| TSGA10 | grey |
| RHOC | grey |
| PSMA2 | grey |
| OCLN | grey |
| FGF6 | grey |
| PID1 | grey |
| SNCG | grey |
| MYCN | grey |
| KIF21B | grey |
| SLC45A4 | grey |
| TBC1D10C | grey |
| DSG3 | grey |
| NPM2 | grey |
| STOX1 | grey |
| SPAG17 | grey |
| TCF23 | grey |
| PIK3R5 | grey |
| GAS2L1 | grey |
| HPDL | grey |
| LRRIQ3 | grey |
| SH3BP4 | grey |
| MPL | grey |
| ITLN1 | grey |
| IL1R1 | grey |
| HABP4 | grey |
| CD3D | grey |
| GSTO1 | grey |
| GZMK | grey |
| C9orf16 | grey |
| TNFSF12 | grey |
| PNLIPRP1 | grey |
| TERT | grey |
| TMCC2 | grey |
| UPB1 | grey |
| SERPINA12 | grey |
| KCNA5 | grey |
| IMMP2L | grey |
| B3GALT5 | grey |
| LRRC7 | grey |
| RAB26 | grey |
| SPP1 | grey |
| EBI3 | grey |
| CLNK | grey |
| AP2S1 | grey |
| UNC5C | grey |
| NID1 | grey |
| NME2 | grey |
| MEOX2 | grey |
| SELENOM | grey |
| CD34 | grey |
| OR52M1 | grey |
| PRICKLE1 | grey |
| MS4A4A | grey |
| DPEP2 | grey |
| IQSEC1 | grey |
| RNF150 | grey |
| ZBP1 | grey |
| RNF128 | grey |
| FGD5 | grey |
| PDCL2 | grey |
| DENND1C | grey |
| CPXM1 | grey |
| VASH2 | grey |
| GNG13 | grey |
| NR2E3 | grey |
| ADTRP | grey |
| FBXL22 | grey |
| ZDHHC14 | grey |
| TSTA3 | grey |
| PRM1 | grey |
| DUSP13 | grey |
| C4BPA | grey |
| GOLGA6L7 | grey |
| LCA5 | grey |
| CD27 | grey |
| GP2 | grey |
| GATA6 | grey |
| LBH | grey |
| KPRP | grey |
| TMIGD3 | grey |
| TUBB2B | grey |
| SNAP25 | grey |
| BGN | grey |
| CLN3 | grey |
| STK32B | grey |
| TLE6 | grey |
| ATP1B1 | grey |
| TNP2 | grey |
| CHRNB4 | grey |
| HHLA2 | grey |
| LINGO2 | grey |
| SLC18A2 | grey |
| CCL3 | grey |
| C19orf33 | grey |
| CACNB4 | grey |
| TREML1 | grey |
| MYOC | grey |
| PCDH15 | grey |
| PLEKHG4B | grey |
| FUT4 | grey |
| SYN2 | grey |
| C16orf46 | grey |
| PLVAP | grey |
| FGF17 | grey |
| ZNF99 | grey |
| LRP6 | grey |
| PLIN4 | grey |
| IL2RG | grey |
| CD300LD | grey |
| CMTM5 | grey |
| PCDHB5 | grey |
| SCGB3A2 | grey |
| GFPT2 | grey |
| CD163 | grey |
| BEND7 | grey |
| DEFA6 | grey |
| DNLZ | grey |
| ADGRF3 | grey |
| RNF225 | grey |
| C4orf3 | grey |
| RARB | grey |
| LLCFC1 | grey |
| C11orf45 | grey |
| NR0B1 | grey |
| NEUROD2 | grey |
| GNA14 | grey |
| ZMAT2 | grey |
| SARNP | grey |
| SOD2 | grey |
| DKK3 | grey |
| BNIP3L | grey |
| TUNAR | grey |
| CYTL1 | grey |
| SORCS1 | grey |
| GLS2 | grey |
| VAMP5 | grey |
| CRABP2 | grey |
| CALML5 | grey |
| FZD6 | grey |
| TMEM212 | grey |
| SLC1A7 | grey |
| NR1I2 | grey |
| APCDD1L | grey |
| ROS1 | grey |
| CYP19A1 | grey |
| OR2D3 | grey |
| PNPLA1 | grey |
| FERMT1 | grey |
| RCAN2 | grey |
| NRG1 | grey |
| CHML | grey |
| TMEM38A | grey |
| PCLO | grey |
| MYOZ2 | grey |
| DSC2 | grey |
| MMP24OS | grey |
| TDRP | grey |
| ELOVL4 | grey |
| OR51Q1 | grey |
| CCM2L | grey |
| ZNF488 | grey |
| HOXD9 | grey |
| ZNF705E | grey |
| CD40LG | grey |
| ZNF711 | grey |
| CXorf58 | grey |
| RHOBTB3 | grey |
| CABLES1 | grey |
| ATOH8 | grey |
| HDAC9 | grey |
| LCE2B | grey |
| HOXA11 | grey |
| BMPR1B | grey |
| FAM163A | grey |
| SALL4 | grey |
| PLD2 | grey |
| ZC3H12C | grey |
| CRABP1 | grey |
| NFKBIB | grey |
| DCD | grey |
| ARPP21 | grey |
| TMEM45B | grey |
| RND1 | grey |
| SHANK3 | grey |
| SLC9A5 | grey |
| MNX1 | grey |
| GJA3 | grey |
| C2CD6 | grey |
| KIR3DL1 | grey |
| RNF10 | grey |
| CAMP | grey |
| HLA-B | grey |
| CDC42BPA | grey |
| CAP2 | grey |
| TAS2R30 | grey |
| HIST1H2AD | grey |
| SLAMF8 | grey |
| SPATC1 | grey |
| ABCA6 | grey |
| ZNF273 | grey |
| NHSL1 | grey |
| TSLP | grey |
| MUC19 | grey |
| ZNF229 | grey |
| PIEZO2 | grey |
| PAGE2B | grey |
| TPI1 | grey |
| FNBP1L | grey |
| FAT1 | grey |
| LEFTY1 | grey |
| FAM186A | grey |
| COL6A6 | grey |
| CCL14 | grey |
| PLIN2 | grey |
| ZFHX3 | grey |
| OR51A7 | grey |
| TAC1 | grey |
| CDH10 | grey |
| BOLL | grey |
| CACNA1I | grey |
| TAFA5 | grey |
| LCN10 | grey |
| KLHDC8B | grey |
| CD84 | grey |
| LPAR2 | grey |
| CPB1 | grey |
| GRID2IP | grey |
| CLIP3 | grey |
| TCAF2 | grey |
| ABI3 | grey |
| MAS1 | grey |
| H1FOO | grey |
| KDM4D | grey |
| LPAR4 | grey |
| LY96 | grey |
| CEACAM6 | grey |
| ACP5 | grey |
| ZAR1L | grey |
| SLC51A | grey |
| LYPD3 | grey |
| GAP43 | grey |
| TET1 | grey |
| PSAPL1 | grey |
| FCRL3 | grey |
| CDRT15 | grey |
| FSTL4 | grey |
| CERCAM | grey |
| SULT1C4 | grey |
| SPRY1 | grey |
| ANKDD1B | grey |
| APOD | grey |
| TMEM63B | grey |
| HCAR1 | grey |
| CLEC2A | grey |
| VNN3 | grey |
| NUCB2 | grey |
| ANKRD31 | grey |
| MTRNR2L8 | grey |
| IFNG | grey |
| CYP27A1 | grey |
| ARHGEF33 | grey |
| EFNA5 | grey |
| GBA | grey |
| XBP1 | grey |
| INSC | grey |
| HOXA10 | grey |
| TENT5A | grey |
| SNX15 | grey |
| SNURF | grey |
| DHX34 | grey |
| CFAP45 | grey |
| PCDHGA3 | grey |
| PCDHA10 | grey |
| TEX12 | grey |
| HIST2H2AC | grey |
| WDR35 | grey |
| BDNF | grey |
| SMAD1 | grey |
| ADRA1D | grey |
| SYNDIG1L | grey |
| ME3 | grey |
| HTR1B | grey |
| SBSN | grey |
| WNK3 | grey |
| PIWIL3 | grey |
| KRT81 | grey |
| KBTBD12 | grey |
| COL6A5 | grey |
| RSPH14 | grey |
| MT2A | grey |
| TCL1A | grey |
| ARID5A | grey |
| SMO | grey |
| GABRB1 | grey |
| OSER1 | grey |
| MAP10 | grey |
| TSPAN9 | grey |
| NTN1 | grey |
| EFHD1 | grey |
| MYRFL | grey |
| MRPL38 | grey |
| PTH2R | grey |
| CBS | grey |
| TPSD1 | grey |
| ROPN1B | grey |
| PCDHB3 | grey |
| RNF112 | grey |
| OR10A2 | grey |
| MSRA | grey |
| CDKL4 | grey |
| SLC22A16 | grey |
| SLC7A7 | grey |
| MMP8 | grey |
| ALDH2 | grey |
| MAEL | grey |
| SYT8 | grey |
| ZBTB10 | grey |
| BST1 | grey |
| CHRDL1 | grey |
| STIMATE | grey |
| SIX3 | grey |
| ZNF536 | grey |
| MGAT1 | grey |
| DEFB1 | grey |
| CD2 | grey |
| EDIL3 | grey |
| TNN | grey |
| TDRD9 | grey |
| BMP1 | grey |
| MYRIP | grey |
| UGT2B10 | grey |
| LAMP5 | grey |
| ARMH4 | grey |
| ZNF729 | grey |
| MAPK4 | grey |
| FFAR3 | grey |
| SYNGR3 | grey |
| SPNS1 | grey |
| FXYD1 | grey |
| FAM124A | grey |
| GAS2L3 | grey |
| PCDHGA4 | grey |
| HS3ST4 | grey |
| KIAA1324 | grey |
| HLA-F | grey |
| PCDHA2 | grey |
| NEK10 | grey |
| PRSS41 | grey |
| AGAP9 | grey |
| CFB | grey |
| ZBTB20 | grey |
| EPB41L4B | grey |
| GXYLT2 | grey |
| COL20A1 | grey |
| CYP4F12 | grey |
| TECRL | grey |
| ZNF676 | grey |
| FRG2C | grey |
| TTC24 | grey |
| INHBB | grey |
| C9orf131 | grey |
| GDPD1 | grey |
| C5 | grey |
| TMEM45A | grey |
| RTEL1 | grey |
| BTNL3 | grey |
| EPHA5 | grey |
| SLC26A9 | grey |
| GRM6 | grey |
| ADAMTS16 | grey |
| GIMAP1 | grey |
| LRFN2 | grey |
| CD48 | grey |
| ACY3 | grey |
| PTGES | grey |
| SLC26A11 | grey |
| DNAH3 | grey |
| NOX4 | grey |
| NTN3 | grey |
| GAD2 | grey |
| ABLIM2 | grey |
| NXF3 | grey |
| PLEKHA6 | grey |
| KIAA0319 | grey |
| CDK11A | grey |
| CEBPA | grey |
| ROBO1 | grey |
| HIST1H2AM | grey |
| TNNT2 | grey |
| ERICH4 | grey |
| C22orf15 | grey |
| GADD45B | grey |
| TBC1D10A | grey |
| DCBLD2 | grey |
| DDI1 | grey |
| CACNG7 | grey |
| C12orf40 | grey |
| AHDC1 | grey |
| RUNX2 | grey |
| C1orf61 | grey |
| PCDHB13 | grey |
| PRSS12 | grey |
| CLDN1 | grey |
| CPA2 | grey |
| KLRC2 | grey |
| BMP5 | grey |
| SNED1 | grey |
| ZFYVE9 | grey |
| FOLR2 | grey |
| IRX6 | grey |
| FAIM2 | grey |
| OVCH1 | grey |
| CAMK1 | grey |
| KRT1 | grey |
| GLUD2 | grey |
| CLSTN2 | grey |
| IL12A | grey |
| CYP17A1 | grey |
| GABARAPL1 | grey |
| AIF1L | grey |
| SLC11A1 | grey |
| TRPA1 | grey |
| ZNF300 | grey |
| PGA3 | grey |
| GNRH2 | grey |
| C1orf141 | grey |
| SLC16A8 | grey |
| JAG1 | grey |
| SLC2A10 | grey |
| LIPI | grey |
| FITM1 | grey |
| RRH | grey |
| MTRNR2L7 | grey |
| CBLN2 | grey |
| SLC5A9 | grey |
| GNG3 | grey |
| UBAP1 | grey |
| COL9A3 | grey |
| CCDC144NL | grey |
| KRAS | grey |
| CPA1 | grey |
| ZMYND15 | grey |
| TIE1 | grey |
| CNMD | grey |
| RASSF5 | grey |
| KIF7 | grey |
| AGRP | grey |
| POU3F3 | grey |
| BEND2 | grey |
| KCTD19 | grey |
| TRAPPC5 | grey |
| LMX1B | grey |
| LIN28A | grey |
| DGKG | grey |
| PLK3 | grey |
| SH2D7 | grey |
| PXT1 | grey |
| GIMAP7 | grey |
| HIST1H3E | grey |
| UPP2 | grey |
| VSTM5 | grey |
| LILRB2 | grey |
| LYVE1 | grey |
| KIRREL1 | grey |
| PHOSPHO1 | grey |
| LIPF | grey |
| OVCH2 | grey |
| ADGRG2 | grey |
| RIMS4 | grey |
| ZC3H12A | grey |
| ATP2C2 | grey |
| SLC8A3 | grey |
| SATB2 | grey |
| CYP4F22 | grey |
| NOP10 | grey |
| NCR3LG1 | grey |
| AMDHD2 | grey |
| RBM11 | grey |
| AXDND1 | grey |
| HIST1H2BJ | grey |
| PLCXD2 | grey |
| TAS2R60 | grey |
| OR52E2 | grey |
| PDE6A | grey |
| AP1S3 | grey |
| TOM1 | grey |
| ALKBH7 | grey |
| CYP1B1 | grey |
| WASHC1 | grey |
| SSC5D | grey |
| WNT2 | grey |
| BOK | grey |
| RAET1E | grey |
| ZNF311 | grey |
| S1PR1 | grey |
| DHRS12 | grey |
| R3HDM4 | grey |
| IL32 | grey |
| NTM | grey |
| HPGD | grey |
| JPH1 | grey |
| ANKRD40CL | grey |
| SUSD4 | grey |
| CHST15 | grey |
| ST8SIA6 | grey |
| SDCBP2 | grey |
| CUX2 | grey |
| ROBO4 | grey |
| MRPS24 | grey |
| NNMT | grey |
| SLC16A2 | grey |
| LSM10 | grey |
| KRTAP5-1 | grey |
| HOXB8 | grey |
| GABRR1 | grey |
| HSPA1A | grey |
| SLITRK4 | grey |
| CAPN3 | grey |
| PWWP3B | grey |
| SLC38A4 | grey |
| TMEM217 | grey |
| TAS2R43 | grey |
| SYNGR1 | grey |
| KCNH3 | grey |
| MAST3 | grey |
| ETS1 | grey |
| ASB5 | grey |
| SH3BP5 | grey |
| RXFP1 | grey |
| EHHADH | grey |
| ANKRD2 | grey |
| GPR160 | grey |
| SLC22A12 | grey |
| HIST2H2AA3 | grey |
| DUSP1 | grey |
| OLFM4 | grey |
| MKNK2 | grey |
| URAD | grey |
| TOMM6 | grey |
| CD8B | grey |
| DEPTOR | grey |
| LTF | grey |
| SYTL4 | grey |
| LIPN | grey |
| PRSS48 | grey |
| OR2W3 | grey |
| ZNF704 | grey |
| NR5A1 | grey |
| CASC3 | grey |
| GLRA1 | grey |
| EYA1 | grey |
| TXNDC2 | grey |
| ZGPAT | grey |
| DIXDC1 | grey |
| PLSCR3 | grey |
| ACSL6 | grey |
| OLAH | grey |
| CSN1S1 | grey |
| HTRA1 | grey |
| PRR32 | grey |
| PECR | grey |
| SHISA9 | grey |
| IFIT3 | grey |
| GPR52 | grey |
| LIMS2 | grey |
| GPC5 | grey |
| GZMH | grey |
| SCARA3 | grey |
| DNAH8 | grey |
| CLEC18C | grey |
| STMN1 | grey |
| HPGDS | grey |
| PYGO1 | grey |
| UGCG | grey |
| CT45A10 | grey |
| GPR3 | grey |
| CD14 | grey |
| ALX3 | grey |
| GIMAP5 | grey |
| COBLL1 | grey |
| IGSF10 | grey |
| HOXB4 | grey |
| RAI2 | grey |
| ALK | grey |
| NUP210L | grey |
| SPAG6 | grey |
| CSNK2B | grey |
| GPM6B | grey |
| TLR10 | grey |
| INO80B | grey |
| ADIPOQ | grey |
| GPR42 | grey |
| BCL2L14 | grey |
| GALR3 | grey |
| GNAI1 | grey |
| OR1J1 | grey |
| PZP | grey |
| MANEAL | grey |
| TUFT1 | grey |
| YPEL3 | grey |
| CLEC4D | grey |
| COCH | grey |
| BLID | grey |
| SLC2A7 | grey |
| ADAMTS18 | grey |
| SFTPC | grey |
| GNG8 | grey |
| APOL4 | grey |
| HLA-A | grey |
| EGLN3 | grey |
| TGM3 | grey |
| CPA3 | grey |
| ZNF662 | grey |
| IFIT2 | grey |
| CACNB2 | grey |
| CCSER1 | grey |
| TNFAIP8L3 | grey |
| OR13D1 | grey |
| DUOX1 | grey |
| SHD | grey |
| PPP2R5B | grey |
| ZFPM2 | grey |
| 2-Mar | grey |
| CHRFAM7A | grey |
| DRD4 | grey |
| PCSK1 | grey |
| FZD8 | grey |
| MANSC4 | grey |
| PTCHD4 | grey |
| HIST1H3D | grey |
| GADD45G | grey |
| PLEKHG6 | grey |
| ZNF334 | grey |
| CDK15 | grey |
| MARCKS | grey |
| PPP1R9A | grey |
| LST1 | grey |
| LRP5 | grey |
| TNFRSF9 | grey |
| PRSS21 | grey |
| ITGA7 | grey |
| CIITA | grey |
| KCNK12 | grey |
| STPG3 | grey |
| CD8B2 | grey |
| DLC1 | grey |
| GATA2 | grey |
| CRLF2 | grey |
| GPR12 | grey |
| ZNF90 | grey |
| PCSK1N | grey |
| HSF5 | grey |
| KHDC1 | grey |
| HOXA5 | grey |
| PRRX1 | grey |
| MLIP | grey |
| XCR1 | grey |
| MORC1 | grey |
| IRF7 | grey |
| MYOM2 | grey |
| OSCP1 | grey |
| OLR1 | grey |
| ESYT3 | grey |
| HRK | grey |
| REELD1 | grey |
| LGALS3 | grey |
| ZNF354C | grey |
| RGS16 | grey |
| FAM221A | grey |
| BMP3 | grey |
| SLC17A9 | grey |
| GALNT15 | grey |
| PCDHB10 | grey |
| B2M | grey |
| SPTBN2 | grey |
| TACR1 | grey |
| AMOTL1 | grey |
| SAT1 | grey |
| AVPR2 | grey |
| IL27 | grey |
| GRIP1 | grey |
| DSP | grey |
| MEDAG | grey |
| GDPD2 | grey |
| TFPI | grey |
| ANO10 | grey |
| BLVRB | grey |
| FCGR1A | grey |
| PABPC4L | grey |
| TMCC3 | grey |
| PTPRD | grey |
| RNASE1 | grey |
| CACNA1C | grey |
| PKD2L1 | grey |
| STXBP5L | grey |
| OR3A1 | grey |
| PROKR1 | grey |
| ITGA9 | grey |
| FSCN1 | grey |
| SH3GL1 | grey |
| UBE2M | grey |
| PLEK2 | grey |
| CCL3L1 | grey |
| WDR45 | grey |
| SOX4 | grey |
| MOSPD3 | grey |
| INS | grey |
| LRRC17 | grey |
| OR52B4 | grey |
| KCNMB3 | grey |
| SPINK9 | grey |
| REG3A | grey |
| UGT2B11 | grey |
| RNF19B | grey |
| CD5 | grey |
| ZAN | grey |
| HIST1H2BE | grey |
| SBK3 | grey |
| SNCAIP | grey |
| FAM221B | grey |
| KRT38 | grey |
| TNFRSF18 | grey |
| LAMA3 | grey |
| TMEM196 | grey |
| CNTFR | grey |
| KCNAB1 | grey |
| OSGIN1 | grey |
| NEGR1 | grey |
| CYP4B1 | grey |
| HOXB7 | grey |
| RGL4 | grey |
| ITGB8 | grey |
| VSTM4 | grey |
| COL22A1 | grey |
| PTGER3 | grey |
| H2AFJ | grey |
| ADCY2 | grey |
| XKR5 | grey |
| CHI3L2 | grey |
| GPR22 | grey |
| FXYD7 | grey |
| SULT2B1 | grey |
| PLA2G5 | grey |
| HIST1H4F | grey |
| OR2AE1 | grey |
| SLC16A9 | grey |
| NUTM1 | grey |
| MXRA5 | grey |
| CILP2 | grey |
| MMP26 | grey |
| FAM214B | grey |
| ZMAT4 | grey |
| ABHD5 | grey |
| ADCY6 | grey |
| SERPINE3 | grey |
| SCN5A | grey |
| SLC35F4 | grey |
| VOPP1 | grey |
| PRSS33 | grey |
| ARAP3 | grey |
| GRIA1 | grey |
| EML5 | grey |
| MIDN | grey |
| NANOS3 | grey |
| HFM1 | grey |
| COL24A1 | grey |
| DPEP2NB | grey |
| OR11L1 | grey |
| NSG2 | grey |
| C10orf82 | grey |
| TRIM71 | grey |
| ZNF365 | grey |
| C1orf100 | grey |
| AMPH | grey |
| SLC23A1 | grey |
| POU4F1 | grey |
| THEM5 | grey |
| KCNE1 | grey |
| INAFM1 | grey |
| OAZ1 | grey |
| PF4 | grey |
| TMEM184A | grey |
| ERRFI1 | grey |
| HOXC5 | grey |
| ILDR2 | grey |
| SOHLH2 | grey |
| XYLB | grey |
| SAGE1 | grey |
| CPT1B | grey |
| FAM107A | grey |
| MYH6 | grey |
| TNFRSF13B | grey |
| C5AR1 | grey |
| TWIST1 | grey |
| PRM2 | grey |
| HOXA7 | grey |
| OR51S1 | grey |
| TTC16 | grey |
| GYG1 | grey |
| BCL3 | grey |
| GATD3A | grey |
| NNAT | grey |
| MPV17L | grey |
| CD37 | grey |
| ANXA8 | grey |
| C17orf98 | grey |
| INSR | grey |
| RHOD | grey |
| CNR1 | grey |
| SYN1 | grey |
| DNMT3A | grey |
| SBK1 | grey |
| DENND5B | grey |
| RXRA | grey |
| CCDC9 | grey |
| KRT14 | grey |
| ISL2 | grey |
| NOX3 | grey |
| MMP27 | grey |
| CCR3 | grey |
| CPXM2 | grey |
| STON2 | grey |
| GLUL | grey |
| UBE2H | grey |
| KIT | grey |
| KCNK13 | grey |
| DMPK | grey |
| MCOLN1 | grey |
| NR5A2 | grey |
| ANGPTL7 | grey |
| CALY | grey |
| RTL8C | grey |
| GIMAP4 | grey |
| TBL1Y | grey |
| POGLUT2 | grey |
| KRTAP5-8 | grey |
| ATRIP | grey |
| TREX1 | grey |
| ALG1L2 | grey |
| OIT3 | grey |
| LTB | grey |
| SERP2 | grey |
| FCN3 | grey |
| KIAA0895 | grey |
| TRPC4 | grey |
| SPON1 | grey |
| CDH4 | grey |
| POLR2J2 | grey |
| SLC17A2 | grey |
| SFTPD | grey |
| HRNR | grey |
| ZNF718 | grey |
| HCAR2 | grey |
| C20orf173 | grey |
| FILIP1 | grey |
| ASGR1 | grey |
| DPF1 | grey |
| SPDYE2B | grey |
| MTRNR2L6 | grey |
| CD79A | grey |
| NAA11 | grey |
| CPA6 | grey |
| SLC3A2 | grey |
| NYAP2 | grey |
| VSIG4 | grey |
| SYT7 | grey |
| BCAS1 | grey |
| ESPN | grey |
| MOBP | grey |
| KIR3DL2 | grey |
| AVPR1B | grey |
| MTRNR2L10 | grey |
| THBD | grey |
| GYPB | grey |
| MEIKIN | grey |
| CGA | grey |
| NAGS | grey |
| KLK2 | grey |
| RNF43 | grey |
| SERPINI2 | grey |
| SLC1A2 | grey |
| OBSL1 | grey |
| S100A8 | grey |
| NOD2 | grey |
| S100A5 | grey |
| NOX5 | grey |
| MAMDC2 | grey |
| ACAP1 | grey |
| TSPAN2 | grey |
| CD59 | grey |
| TMPRSS11A | grey |
| ANPEP | grey |
| CPNE5 | grey |
| DUSP8 | grey |
| DNAH14 | grey |
| RIPPLY3 | grey |
| PCDHGB3 | grey |
| TACR2 | grey |
| PKDREJ | grey |
| RBP7 | grey |
| NXPH3 | grey |
| CYP2W1 | grey |
| KCNJ14 | grey |
| HAND2 | grey |
| TSSK3 | grey |
| DRD5 | grey |
| CEACAM8 | grey |
| ARMC12 | grey |
| TUBB3 | grey |
| USP17L2 | grey |
| BAIAP3 | grey |
| HILPDA | grey |
| SKIDA1 | grey |
| CD1A | grey |
| APOA4 | grey |
| CDH19 | grey |
| ZNF462 | grey |
| OR51E2 | grey |
| IL1RL2 | grey |
| NFE4 | grey |
| OR13C3 | grey |
| CASKIN2 | grey |
| FAM209A | grey |
| PLD1 | grey |
| KLHL38 | grey |
| RD3 | grey |
| AK1 | grey |
| HAMP | grey |
| GPR173 | grey |
| ARG1 | grey |
| ZBED8 | grey |
| BAG3 | grey |
| TSPAN5 | grey |
| COL2A1 | grey |
| COL23A1 | grey |
| CBX7 | grey |
| ITPKA | grey |
| LDHAL6B | grey |
| C19orf57 | grey |
| SPSB3 | grey |
| LAMB2 | grey |
| P4HA3 | grey |
| CD163L1 | grey |
| C9 | grey |
| KLRB1 | grey |
| RTP3 | grey |
| C11orf52 | grey |
| VSIG8 | grey |
| MTUS2 | grey |
| GSDMA | grey |
| SLC9C1 | grey |
| SOX1 | grey |
| MEGF10 | grey |
| KLHDC8A | grey |
| CBX2 | grey |
| SIGLEC10 | grey |
| ENKUR | grey |
| TMEM200B | grey |
| HIST1H2BH | grey |
| FIBIN | grey |
| FIGN | grey |
| SEL1L2 | grey |
| KIF19 | grey |
| GATA4 | grey |
| RET | grey |
| KCNN2 | grey |
| CDC42EP2 | grey |
| EPHB1 | grey |
| MPP6 | grey |
| PRSS37 | grey |
| ST18 | grey |
| SEMG1 | grey |
| ERVFRD-1 | grey |
| CTSS | grey |
| PCDHGA8 | grey |
| FDCSP | grey |
| MATN4 | grey |
| GREM1 | grey |
| PLA2G7 | grey |
| BCL7A | grey |
| FAM166A | grey |
| CCDC27 | grey |
| INSYN2B | grey |
| C3orf80 | grey |
| GNGT2 | grey |
| OR52R1 | grey |
| EIF4A1 | grey |
| SH3PXD2A | grey |
| CEBPB | grey |
| MCMDC2 | grey |
| ACOT11 | grey |
| NXPH4 | grey |
| ARHGAP45 | grey |
| RUBCNL | grey |
| CALN1 | grey |
| CNKSR3 | grey |
| MEIS2 | grey |
| NT5DC4 | grey |
| HHAT | grey |
| BEX5 | grey |
| NKAIN3 | grey |
| OPRK1 | grey |
| HKDC1 | grey |
| MUSTN1 | grey |
| RTL1 | grey |
| DENND2D | grey |
| ZFR2 | grey |
| GAREM1 | grey |
| HMX2 | grey |
| OR51B6 | grey |
| CALB1 | grey |
| GNG11 | grey |
| SCT | grey |
| MMRN1 | grey |
| LRGUK | grey |
| ZNF793 | grey |
| HPSE | grey |
| EPX | grey |
| OR2G3 | grey |
| FAM111B | grey |
| OR2C1 | grey |
| SLC2A12 | grey |
| SLC26A1 | grey |
| NANOS1 | grey |
| MYLK4 | grey |
| DNAJC28 | grey |
| HACD1 | grey |
| ITGA2 | grey |
| MAP2K3 | grey |
| CDK5R1 | grey |
| CCL7 | grey |
| RAB19 | grey |
| RSPH6A | grey |
| KCNK10 | grey |
| SP140 | grey |
| EVPL | grey |
| DNAJB1 | grey |
| FCGR1B | grey |
| ARMCX1 | grey |
| SEC14L1 | grey |
| ATF7IP2 | grey |
| PCOLCE2 | grey |
| CCDC88B | grey |
| PDX1 | grey |
| MC4R | grey |
| EFEMP1 | grey |
| CT45A5 | grey |
| WNT8B | grey |
| GPR146 | grey |
| ACCS | grey |
| RNF182 | grey |
| OR2L5 | grey |
| SFTPB | grey |
| CRISP2 | grey |
| EXOSC4 | grey |
| CTSL | grey |
| AMZ1 | grey |
| CHRNA1 | grey |
| CNTN1 | grey |
| CLDN5 | grey |
| NYNRIN | grey |
| MLNR | grey |
| GLTP | grey |
| PCDHB15 | grey |
| FAM71D | grey |
| MROH7 | grey |
| ADAM21 | grey |
| LINC02218 | grey |
| NR2E1 | grey |
| TRARG1 | grey |
| SLITRK5 | grey |
| LRRC74A | grey |
| MEX3A | grey |
| DOCK1 | grey |
| RAB3IL1 | grey |
| KIR3DL3 | grey |
| ZSCAN23 | grey |
| BCKDK | grey |
| ARHGAP22 | grey |
| TPBG | grey |
| CELF3 | grey |
| FLNB | grey |
| HOXB5 | grey |
| CES3 | grey |
| TAFA2 | grey |
| KLHL29 | grey |
| KIF17 | grey |
| DEFA3 | grey |
| WNT7B | grey |
| OR52K2 | grey |
| UPK3B | grey |
| SSTR2 | grey |
| IFIT1 | grey |
| FZD7 | grey |
| MDFI | grey |
| OSTN | grey |
| IRX5 | grey |
| SLC25A47 | grey |
| MMP16 | grey |
| ALX4 | grey |
| SMIM18 | grey |
| OR10H1 | grey |
| WNT7A | grey |
| LSMEM1 | grey |
| PCDHGA2 | grey |
| OR1N2 | grey |
| TMEM176B | grey |
| TRPM6 | grey |
| PRLR | grey |
| SEZ6 | grey |
| PHACTR3 | grey |
| TAS2R9 | grey |
| KLRF1 | grey |
| AOC2 | grey |
| MT1H | grey |
| OR14A16 | grey |
| GCM1 | grey |
| CELA2A | grey |
| NIPA1 | grey |
| NATD1 | grey |
| UGT2B28 | grey |
| PIWIL4 | grey |
| VWC2L | grey |
| MAP3K21 | grey |
| HBG1 | grey |
| GPR84 | grey |
| CCDC179 | grey |
| CEACAM16 | grey |
| TMUB1 | grey |
| CACNA1E | grey |
| HIST3H3 | grey |
| TSPAN10 | grey |
| CRISPLD1 | grey |
| NFIL3 | grey |
| NGB | grey |
| SCN7A | grey |
| ETV7 | grey |
| INSYN2A | grey |
| PCDH1 | grey |
| ARL5C | grey |
| TCEAL2 | grey |
| KIR2DL3 | grey |
| PVALB | grey |
| GBP1 | grey |
| C11orf95 | grey |
| BMP6 | grey |
| CDH11 | grey |
| LCN6 | grey |
| KLRC3 | grey |
| PPP1R1B | grey |
| HPSE2 | grey |
| CHD5 | grey |
| GPR87 | grey |
| CHRM5 | grey |
| KRT9 | grey |
| C3orf56 | grey |
| OR51G1 | grey |
| FAM81A | grey |
| FUT9 | grey |
| PCDHGB7 | grey |
| CXCR4 | grey |
| PCDHB2 | grey |
| HES4 | grey |
| NRN1L | grey |
| LHX1 | grey |
| UBASH3A | grey |
| PCDHGA7 | grey |
| CEMIP | grey |
| PTPRN | grey |
| LTA | grey |
| CAPS2 | grey |
| CEACAM3 | grey |
| COX6B2 | grey |
| PLEKHG3 | grey |
| ENO4 | grey |
| ADGRE3 | grey |
| CYP21A2 | grey |
| PSMB10 | grey |
| CLDN10 | grey |
| ANO7 | grey |
| ZFHX2 | grey |
| DBNDD2 | grey |
| PRDM16 | grey |
| PTN | grey |
| CPZ | grey |
| KIAA1522 | grey |
| EFR3B | grey |
| FAM110C | grey |
| PTRH1 | grey |
| FCN2 | grey |
| CIDEC | grey |
| LCN2 | grey |
| ADAM32 | grey |
| SPOCD1 | grey |
| GLB1L3 | grey |
| C5orf67 | grey |
| NRGN | grey |
| SH2B2 | grey |
| ETNPPL | grey |
| NLRP11 | grey |
| SLC22A18 | grey |
| NOG | grey |
| MALRD1 | grey |
| PLXNB1 | grey |
| OR52A1 | grey |
| PLA2G2F | grey |
| GARNL3 | grey |
| LYPD2 | grey |
| FGF1 | grey |
| RANBP3L | grey |
| GDI1 | grey |
| ZNF683 | grey |
| RBM24 | grey |
| LRAT | grey |
| KCNMA1 | grey |
| GCK | grey |
| PEX5L | grey |
| MUCL3 | grey |
| FNDC8 | grey |
| KDM4E | grey |
| KIAA1217 | grey |
| GPR17 | grey |
| PCDH10 | grey |
| TLE3 | grey |
| CCDC148 | grey |
| CLEC6A | grey |
| EEF1G | grey |
| MPP3 | grey |
| FTL | grey |
| DCAF12 | grey |
| ANKRD34B | grey |
| TDRD15 | grey |
| PADI4 | grey |
| JAK2 | grey |
| SIT1 | grey |
| AKR1C2 | grey |
| GRIK4 | grey |
| UBE2V1 | grey |
| FTCDNL1 | grey |
| CYP11A1 | grey |
| HAPLN3 | grey |
| DCN | grey |
| B3GALT1 | grey |
| TRAF1 | grey |
| OR2G2 | grey |
| GPC4 | grey |
| CTSH | grey |
| CLDN11 | grey |
| BEND6 | grey |
| MYEOV | grey |
| SLC43A2 | grey |
| OR3A2 | grey |
| CACNG4 | grey |
| HOXA4 | grey |
| NUTM2F | grey |
| TMEM238L | grey |
| DLL3 | grey |
| PRMT8 | grey |
| FAM53C | grey |
| PADI3 | grey |
| MYL6 | grey |
| HCRTR1 | grey |
| SPOCK3 | grey |
| ANKFN1 | grey |
| SALL1 | grey |
| PCDHGC4 | grey |
| TRDN | grey |
| NPNT | grey |
| CNRIP1 | grey |
| GRIFIN | grey |
| VANGL1 | grey |
| NMU | grey |
| NELL2 | grey |
| MDGA2 | grey |
| HECW2 | grey |
| OTX1 | grey |
| TRAFD1 | grey |
| CALCRL | grey |
| NECAB2 | grey |
| MTRNR2L5 | grey |
| TLCD1 | grey |
| RAP1GAP | grey |
| CEP70 | grey |
| MARVELD2 | grey |
| PRDX5 | grey |
| IPO4 | grey |
| COX8A | grey |
| COL6A2 | grey |
| GOLGA8M | grey |
| GOLGA8O | grey |
| CTRC | grey |
| IRGM | grey |
| TBX1 | grey |
| LEP | grey |
| ADGRL3 | grey |
| PNLIP | grey |
| CLEC18A | grey |
| SLC9A4 | grey |
| HPCA | grey |
| MAF | grey |
| ADCY1 | grey |
| SMIM1 | grey |
| CAMK2N1 | grey |
| AMH | grey |
| CHMP4B | grey |
| OR2L3 | grey |
| GCSAML | grey |
| HIST1H4C | grey |
| KRT71 | grey |
| EMID1 | grey |
| PGC | grey |
| USP43 | grey |
| RGS10 | grey |
| TBC1D3D | grey |
| TRADD | grey |
| AVPI1 | grey |
| PFKFB2 | grey |
| AS3MT | grey |
| CTRB2 | grey |
| ARHGAP32 | grey |
| BAHCC1 | grey |
| TGM5 | grey |
| NCAM2 | grey |
| ZCCHC12 | grey |
| NKX2-3 | grey |
| AP1M2 | grey |
| GOLGA6L9 | grey |
| ARHGAP36 | grey |
| NARF | grey |
| SDS | grey |
| TBR1 | grey |
| CLIP2 | grey |
| SLC10A5 | grey |
| ITPRIP | grey |
| ANXA5 | grey |
| BTNL8 | grey |
| PCDHB8 | grey |
| OR51E1 | grey |
| OR2L2 | grey |
| PLCH1 | grey |
| TCF4 | grey |
| RAB7B | grey |
| CXCL5 | grey |
| KCNH1 | grey |
| HOXB9 | grey |
| TCEA3 | grey |
| DUSP19 | grey |
| ALPP | grey |
| SCN11A | grey |
| JMJD6 | grey |
| IGF2R | grey |
| VEGFA | grey |
| SLC15A4 | grey |
| DDIT3 | grey |
| CARD19 | grey |
| DNAJC12 | grey |
| GATD3B | grey |
| CTTN | grey |
| SLC6A2 | grey |
| TSSC4 | grey |
| GGT5 | grey |
| ASIP | grey |
| ZAR1 | grey |
| HHIPL1 | grey |
| COL11A1 | grey |
| GPT2 | grey |
| NPRL3 | grey |
| GNLY | grey |
| TCIM | grey |
| VWA1 | grey |
| CDH6 | grey |
| MAPK12 | grey |
| ACSS3 | grey |
| RTL9 | grey |
| ATP7B | grey |
| HDAC10 | grey |
| NINJ1 | grey |
| RNF166 | grey |
| OR2C3 | grey |
| TRIM39 | grey |
| LCE5A | grey |
| TLL2 | grey |
| HTR7 | grey |
| RHBDL3 | grey |
| STOM | grey |
| ADGRA2 | grey |
| ENTPD8 | grey |
| PDZK1IP1 | grey |
| GABRA6 | grey |
| IP6K3 | grey |
| HHIP | grey |
| EMD | grey |
| PELO | grey |
| TNIP1 | grey |
| PHKA1 | grey |
| OR52J3 | grey |
| PCDH18 | grey |
| MUC6 | grey |
| SAP25 | grey |
| NUDT10 | grey |
| TTLL8 | grey |
| PCDHGA1 | grey |
| KCNK1 | grey |
| ZNF157 | grey |
| TMEM204 | grey |
| HOXA3 | grey |
| TCTEX1D1 | grey |
| STPG2 | grey |
| FLT3 | grey |
| PATE4 | grey |
| PTPRZ1 | grey |
| C10orf95 | grey |
| SP110 | grey |
| PRG2 | grey |
| ADAMTS20 | grey |
| CYP27C1 | grey |
| RHOBTB1 | grey |
| CMYA5 | grey |
| VMO1 | grey |
| OSR1 | grey |
| KRTDAP | grey |
| NLRP9 | grey |
| ZNF214 | grey |
| C5orf63 | grey |
| PCDHA3 | grey |
| PAMR1 | grey |
| SCN2A | grey |
| OR1L8 | grey |
| SPRY2 | grey |
| IL31 | grey |
| VWA2 | grey |
| AMN | grey |
| CALB2 | grey |
| GADD45A | grey |
| CEACAM21 | grey |
| IL1RN | grey |
| COL14A1 | grey |
| PELI1 | grey |
| EPHA3 | grey |
| RLBP1 | grey |
| CD24 | grey |
| INSL6 | grey |
| TXNIP | grey |
| COL4A5 | grey |
| CACNG6 | grey |
| NRG4 | grey |
| PINK1 | grey |
| TENM4 | grey |
| CDH2 | grey |
| RNASE4 | grey |
| CLEC4A | grey |
| TBX10 | grey |
| KCNIP1 | grey |
| ARID3B | grey |
| PREX2 | grey |
| PPAN | grey |
| TSPO2 | grey |
| TNC | grey |
| FCGR3B | grey |
| NUDT12 | grey |
| EPGN | grey |
| CHRNG | grey |
| OR56A1 | grey |
| KLF2 | grey |
| MAGEC3 | grey |
| COL13A1 | grey |
| REM2 | grey |
| ENTPD2 | grey |
| APBA1 | grey |
| PF4V1 | grey |
| PNRC1 | grey |
| LRWD1 | grey |
| RIBC2 | grey |
| OR13F1 | grey |
| NFKB2 | grey |
| AQP10 | grey |
| OR52B6 | grey |
| KLF1 | grey |
| MRGPRX2 | grey |
| XCL2 | grey |
| CCK | grey |
| TAFA3 | grey |
| CHRM3 | grey |
| FAM83H | grey |
| ARL6IP4 | grey |
| MAP2K6 | grey |
| TMEM171 | grey |
| CCN1 | grey |
| MYCBPAP | grey |
| LFNG | grey |
| ZC2HC1C | grey |
| PTGES3L | grey |
| BSG | grey |
| SLC2A14 | grey |
| METTL21C | grey |
| HOXB1 | grey |
| ZSCAN1 | grey |
| MTRNR2L1 | grey |
| WBP2 | grey |
| SCGB2A2 | grey |
| SLC35G3 | grey |
| LGALS9C | grey |
| CA1 | grey |
| APOL2 | grey |
| STBD1 | grey |
| ICAM1 | grey |
| ZNF804A | grey |
| PLA2G4B | grey |
| DOCK4 | grey |
| DLGAP2 | grey |
| ICOS | grey |
| RASSF8 | grey |
| C10orf105 | grey |
| MYL4 | grey |
| CYP4F3 | grey |
| RAB20 | grey |
| DIPK1B | grey |
| ZNF385C | grey |
| SHF | grey |
| KRT77 | grey |
| WDR49 | grey |
| SDK2 | grey |
| SOX12 | grey |
| EREG | grey |
| U2AF1 | grey |
| CALML3 | grey |
| CIDEA | grey |
| HSPG2 | grey |
| COL9A2 | grey |
| RGS9 | grey |
| ACSM6 | grey |
| MRPL12 | grey |
| BFSP1 | grey |
| CORO7 | grey |
| PRTG | grey |
| EHD2 | grey |
| PRRG1 | grey |
| LRRC6 | grey |
| NMNAT2 | grey |
| BLK | grey |
| TMSB15B | grey |
| FAM83B | grey |
| LSAMP | grey |
| TRMT9B | grey |
| HSPA6 | grey |
| FMNL1 | grey |
| DPYSL3 | grey |
| LRBA | grey |
| PPIL6 | grey |
| LY6G6F | grey |
| CRIM1 | grey |
| FABP2 | grey |
| DHRS9 | grey |
| VWDE | grey |
| GPNMB | grey |
| TEX53 | grey |
| TRPC5OS | grey |
| DEPDC7 | grey |
| ZNRF1 | grey |
| NEFL | grey |
| MYT1L | grey |
| CREM | grey |
| CD200 | grey |
| PALD1 | grey |
| CFAP47 | grey |
| C1orf68 | grey |
| LOR | grey |
| LRRC8E | grey |
| HK2 | grey |
| DYNAP | grey |
| CEMIP2 | grey |
| GKN1 | grey |
| NPIPA2 | grey |
| USP44 | grey |
| FOXL1 | grey |
| CCR6 | grey |
| COL7A1 | grey |
| SLC22A1 | grey |
| LY6G5B | grey |
| CCR7 | grey |
| STOX2 | grey |
| ADRA2A | grey |
| CLUL1 | grey |
| FGF10 | grey |
| BRSK1 | grey |
| WDR90 | grey |
| TNP1 | grey |
| METTL9 | grey |
| SERINC2 | grey |
| FAM227A | grey |
| SCHIP1 | grey |
| CLDN19 | grey |
| HPCAL4 | grey |
| GLRA4 | grey |
| MYH7B | grey |
| UBXN1 | grey |
| TIFAB | grey |
| CNTN5 | grey |
| SPINK4 | grey |
| ERAS | grey |
| TREML4 | grey |
| FSIP2 | grey |
| TSBP1 | grey |
| FSD1 | grey |
| WBP2NL | grey |
| RBFOX1 | grey |
| BBC3 | grey |
| FIBCD1 | grey |
| TOM1L1 | grey |
| ATP1A2 | grey |
| SLC6A3 | grey |
| MIF | grey |
| IFRD1 | grey |
| FAM205A | grey |
| SHH | grey |
| LRRC31 | grey |
| SCUBE3 | grey |
| KRT86 | grey |
| GPR78 | grey |
| CPLX3 | grey |
| CCRL2 | grey |
| TNS1 | grey |
| SIAH2 | grey |
| TNFRSF14 | grey |
| GNAZ | grey |
| MYO5C | grey |
| RPA4 | grey |
| SEPTIN4 | grey |
| SEZ6L | grey |
| NBEA | grey |
| TPSG1 | grey |
| ASIC1 | grey |
| RDH12 | grey |
| RBM38 | grey |
| FZD9 | grey |
| FGF9 | grey |
| KIR2DL1 | grey |
| GLRX5 | grey |
| ARL17A | grey |
| SEC14L5 | grey |
| SLC5A4 | grey |
| PPP1R27 | grey |
| CCDC124 | grey |
| TMEM100 | grey |
| OR56A3 | grey |
| PNPLA6 | grey |
| PPARD | grey |
| TGFB1 | grey |
| SLC44A5 | grey |
| TTLL6 | grey |
| GOLGA8Q | grey |
| PLA2G4F | grey |
| S100P | grey |
| OVOL3 | grey |
| CNTNAP5 | grey |
| CCL21 | grey |
| HBQ1 | grey |
| GPR15 | grey |
| SMIM2 | grey |
| ZNF469 | grey |
| KLRD1 | grey |
| ATP13A4 | grey |
| SOD3 | grey |
| BOLA2 | grey |
| CRP | grey |
| OR51M1 | grey |
| DIPK1C | grey |
| AHNAK2 | grey |
| KCNK5 | grey |
| P2RY1 | grey |
| KRT73 | grey |
| SLC16A6 | grey |
| PIM3 | grey |
| 2-Mar | grey |
| CHST4 | grey |
| C16orf90 | grey |
| TNFRSF13C | grey |
| SUPT3H | grey |
| LRRC18 | grey |
| C1QA | grey |
| TCN1 | grey |
| CD28 | grey |
| GPS2 | grey |
| RRAD | grey |
| TREH | grey |
| HDC | grey |
| PRKD2 | grey |
| ADAMTS6 | grey |
| MT1E | grey |
| KRT23 | grey |
| MELTF | grey |
| SYCN | grey |
| ANKRD18A | grey |
| PVALEF | grey |
| FGF16 | grey |
| GFAP | grey |
| GBP2 | grey |
| HMGA2 | grey |
| CASP14 | grey |
| CRB2 | grey |
| ZBTB8A | grey |
| PFDN4 | grey |
| CAGE1 | grey |
| TSPOAP1 | grey |
| C1orf116 | grey |
| AP3B2 | grey |
| MMP9 | grey |
| OR51B2 | grey |
| GYG2 | grey |
| LMO1 | grey |
| SAA2 | grey |
| LRRIQ4 | grey |
| CBSL | grey |
| ADGRE1 | grey |
| C4orf48 | grey |
| FLRT3 | grey |
| PCDHGA11 | grey |
| SAA1 | grey |
| ANKRD63 | grey |
| CD6 | grey |
| SULT1A4 | grey |
| GPSM1 | grey |
| NPFFR1 | grey |
| KLHL13 | grey |
| GZMA | grey |
| SAMD11 | grey |
| BDKRB1 | grey |
| F5 | grey |
| PCDHGA5 | grey |
| KAZN | grey |
| MICU3 | grey |
| SYTL3 | grey |
| TCL1B | grey |
| CDKN2D | grey |
| HPN | grey |
| CSMD3 | grey |
| KCNE5 | grey |
| CPE | grey |
| GPD1 | grey |
| PCDHGA10 | grey |
| IQSEC3 | grey |
| ABCA12 | grey |
| MT1G | grey |
| PI16 | grey |
| RNF144B | grey |
| RFX8 | grey |
| FOXN1 | grey |
| SP7 | grey |
| SLC46A2 | grey |
| THAP10 | grey |
| DNER | grey |
| IER3 | grey |
| SORBS2 | grey |
| ST8SIA3 | grey |
| PPP1R15A | grey |
| DKK2 | grey |
| IRF9 | grey |
| DEGS2 | grey |
| SH2D3A | grey |
| TUBB2A | grey |
| HIGD1C | grey |
| LRIT3 | grey |
| ARHGAP5 | grey |
| THBS1 | grey |
| GMPR | grey |
| CNTNAP3B | grey |
| SLX1A | grey |
| GRM7 | grey |
| ZNF454 | grey |
| C3orf67 | grey |
| CHST7 | grey |
| SLITRK6 | grey |
| CLGN | grey |
| C14orf132 | grey |
| NRXN2 | grey |
| ARMC9 | grey |
| SEMA6B | grey |
| MS4A2 | grey |
| GBP6 | grey |
| CTRL | grey |
| CD109 | grey |
| HSPB1 | grey |
| S100A10 | grey |
| LAPTM4B | grey |
| PGLYRP1 | grey |
| TTC4 | grey |
| PPDPF | grey |
| ALDH1A2 | grey |
| GLIPR1L1 | grey |
| TIMP1 | grey |
| WASF1 | grey |
| SLC45A3 | grey |
| TMEM72 | grey |
| MYBPC3 | grey |
| TMEM176A | grey |
| KLRG2 | grey |
| LCT | grey |
| RAB36 | grey |
| CFAP58 | grey |
| FCER1A | grey |
| UBE2F | grey |
| KLK5 | grey |
| C19orf18 | grey |
| CELF6 | grey |
| RAG2 | grey |
| HMOX1 | grey |
| RS1 | grey |
| AQP1 | grey |
| MIP | grey |
| OASL | grey |
| TAS2R41 | grey |
| CLEC7A | grey |
| HEPHL1 | grey |
| FAM9B | grey |
| HIST3H2BB | grey |
| ZFAND2B | grey |
| ANKRD33 | grey |
| LRP11 | grey |
| HCAR3 | grey |
| CDH9 | grey |
| C18orf32 | grey |
| GOLGA8R | grey |
| AMACR | grey |
| CD38 | grey |
| MT1M | grey |
| ITGAD | grey |
| AIM2 | grey |
| CLEC1A | grey |
| DDO | grey |
| C17orf49 | grey |
| APBB1 | grey |
| TMEM158 | grey |
| INPP5K | grey |
| HBM | grey |
| CFAP69 | grey |
| SLCO5A1 | grey |
| TSC22D3 | grey |
| TAS2R42 | grey |
| SH3D19 | grey |
| PCDHGB1 | grey |
| RHOB | grey |
| PGA5 | grey |
| SLC22A11 | grey |
| SV2C | grey |
| SLA2 | grey |
| TSPYL2 | grey |
| CCDC151 | grey |
| INMT | grey |
| SLAMF7 | grey |
| ABLIM3 | grey |
| NEURL1B | grey |
| DYTN | grey |
| ASGR2 | grey |
| HIST2H2AA4 | grey |
| CNGB3 | grey |
| HP | grey |
| MEX3B | grey |
| AICDA | grey |
| FAM20A | grey |
| CSRNP1 | grey |
| CCDC144A | grey |
| MAP2 | grey |
| DRAXIN | grey |
| GSTA1 | grey |
| LOX | grey |
| STARD10 | grey |
| CTLA4 | grey |
| USP17L7 | grey |
| FLRT1 | grey |
| FAM124B | grey |
| ALDH1L2 | grey |
| CAPN5 | grey |
| SMCO2 | grey |
| C1QB | grey |
| APOLD1 | grey |
| SFRP2 | grey |
| LY9 | grey |
| MYCT1 | grey |
| FOXC1 | grey |
| GJA9 | grey |
| KLHL33 | grey |
| SEMA3A | grey |
| DCHS1 | grey |
| UGT3A2 | grey |
| IFNK | grey |
| TCF21 | grey |
| PLAAT1 | grey |
| PODN | grey |
| JMJD7 | grey |
| RGS9BP | grey |
| MPP1 | grey |
| AKNAD1 | grey |
| LOXL4 | grey |
| ZNF560 | grey |
| BCL2A1 | grey |
| ANKHD1 | grey |
| PCDHGB6 | grey |
| CHRNA6 | grey |
| PTGDR | grey |
| CDHR1 | grey |
| SYT2 | grey |
| ADCYAP1R1 | grey |
| LGALSL | grey |
| CSRP2 | grey |
| ZNF107 | grey |
| CSMD1 | grey |
| FRMD6 | grey |
| HEYL | grey |
| ADAM28 | grey |
| FABP6 | grey |
| SAXO2 | grey |
| MSTN | grey |
| RGL3 | grey |
| SRSF12 | grey |
| ANKRD22 | grey |
| HSD11B1 | grey |
| MPO | grey |
| IL7R | grey |
| NAV3 | grey |
| SLC10A6 | grey |
| PTPN3 | grey |
| PXYLP1 | grey |
| CXCR5 | grey |
| NAALADL2 | grey |
| SMAD7 | grey |
| MMP15 | grey |
| TMEM40 | grey |
| NUCB1 | grey |
| TOX2 | grey |
| RYR3 | grey |
| OR51T1 | grey |
| RAMP3 | grey |
| RTP5 | grey |
| PDZD2 | grey |
| LIM2 | grey |
| TBC1D30 | grey |
| SYCE3 | grey |
| JAKMIP1 | grey |
| CASP5 | grey |
| HSD3B2 | grey |
| BRINP2 | grey |
| SYNGR2 | grey |
| FAM241B | grey |
| CDH23 | grey |
| FBXO2 | grey |
| CRYBG3 | grey |
| SUCNR1 | grey |
| CYP2A7 | grey |
| CYP2B6 | grey |
| HIST1H3H | grey |
| RASGRP3 | grey |
| SNTB2 | grey |
| DNAH6 | grey |
| HLA-G | grey |
| DNAH11 | grey |
| COL18A1 | grey |
| PTPRG | grey |
| ACSM1 | grey |
| ZNF215 | grey |
| GCM2 | grey |
| PTPN14 | grey |
| ALPK2 | grey |
| PTK7 | grey |
| ZNF112 | grey |
| OSBP2 | grey |
| GPT | grey |
| ZNF804B | grey |
| BEND4 | grey |
| TIMP3 | grey |
| GUCY1B1 | grey |
| CYP46A1 | grey |
| TWISTNB | grey |
| ACTL8 | grey |
| ACSM3 | grey |
| SH3TC2 | grey |
| APC2 | grey |
| RCN3 | grey |
| ZNF534 | grey |
| CHRNA5 | grey |
| OR51F1 | grey |
| F2RL2 | grey |
| RNF217 | grey |
| PSD3 | grey |
| HOXA6 | grey |
| NCR2 | grey |
| HECTD2 | grey |
| ERVMER34-1 | grey |
| RGS6 | grey |
| ANKRD34A | grey |
| GSG1 | grey |
| WT1 | grey |
| HIST1H2BK | grey |
| OPHN1 | grey |
| RADIL | grey |
| IKZF2 | grey |
| SPIRE1 | grey |
| TKTL1 | grey |
| MEGF11 | grey |
| CNPPD1 | grey |
| FAM155B | grey |
| MT1A | grey |
| HTR3A | grey |
| MGP | grey |
| SRGAP3 | grey |
| CSTB | grey |
| SPNS2 | grey |
| C22orf23 | grey |
| SMC1B | grey |
| FLT3LG | grey |
| ZNF728 | grey |
| CUEDC1 | grey |
| UNC119 | grey |
| LPO | grey |
| VNN1 | grey |
| RASA4B | grey |
| NKAIN2 | grey |
| MECOM | grey |
| SIGLEC7 | grey |
| CCR4 | grey |
| VKORC1 | grey |
| TPSAB1 | grey |
| CBY3 | grey |
| KLF14 | grey |
| DNAJB13 | grey |
| LRRD1 | grey |
| CYP3A7 | grey |
| MT1F | grey |
| OPLAH | grey |
| NEUROD1 | grey |
| SLC6A12 | grey |
| TPTE2 | grey |
| ANKRD18B | grey |
| RENBP | grey |
| RPP21 | grey |
| BHLHE22 | grey |
| SMARCD3 | grey |
| IRX1 | grey |
| POM121L2 | grey |
| CD300A | grey |
| EDNRB | grey |
| CTSB | grey |
| TXNRD3 | grey |
| CPNE7 | grey |
| SMOX | grey |
| HIST1H2AC | grey |
| RHEX | grey |
| ZNF165 | grey |
| GPR37L1 | grey |
| CABP5 | grey |
| FHL5 | grey |
| HIST1H2BC | grey |
| SH3RF2 | grey |
| BNC1 | grey |
| C11orf94 | grey |
| MED12L | grey |
| PIK3IP1 | grey |
| PCDHGB5 | grey |
| LILRA1 | grey |
| PURG | grey |
| MAGEA12 | grey |
| C20orf203 | grey |
| MSI2 | grey |
| RORC | grey |
| MYEF2 | grey |
| GH1 | grey |
| MOB1B | grey |
| SPINK8 | grey |
| TNFRSF19 | grey |
| HIST1H2AL | grey |
| KCTD1 | grey |
| AFF2 | grey |
| RNF157 | grey |
| ID3 | grey |
| KRT31 | grey |
| SIGLEC6 | grey |
| TRIM63 | grey |
| IGDCC4 | grey |
| DIRAS2 | grey |
| TTC22 | grey |
| HGF | grey |
| OR2AG2 | grey |
| RGS4 | grey |
| CCL4L2 | grey |
| ACTC1 | grey |
| GPBAR1 | grey |
| RTL3 | grey |
| SLAMF1 | grey |
| PLCB4 | grey |
| RASAL2 | grey |
| BHLHE40 | grey |
| OR10A4 | grey |
| GRIK1 | grey |
| CYSTM1 | grey |
| ZNF23 | grey |
| GDPD3 | grey |
| CGREF1 | grey |
| DACT2 | grey |
| IL10RA | grey |
| TBX4 | grey |
| THSD7B | grey |
| PPP1R3A | grey |
| UBALD2 | grey |
| PTCRA | grey |
| FAIM | grey |
| CTHRC1 | grey |
| JAZF1 | grey |
| IFITM3 | grey |
| TAF7L | grey |
| PARD6A | grey |
| ZNF208 | grey |
| SLC25A37 | grey |
| DEUP1 | grey |
| LDLRAD2 | grey |
| SLC5A12 | grey |
| C6orf226 | grey |
| KCNG1 | grey |
| SAT2 | grey |
| FAM171A1 | grey |
| USH2A | grey |
| OR2L13 | grey |
| IGSF21 | grey |
| CYP4F11 | grey |
| PRG3 | grey |
| SLC9A3 | grey |
| LAMB3 | grey |
| RCOR2 | grey |
| ITGB5 | grey |
| CEBPE | grey |
| BPI | grey |
| MYOZ3 | grey |
| OR51G2 | grey |
| SPANXB1 | grey |
| GOLGA8T | grey |
| INSL3 | grey |
| EHBP1L1 | grey |
| TNS3 | grey |
| CC2D2A | grey |
| PDXP | grey |
| OR6A2 | grey |
| TSHR | grey |
| TFF1 | grey |
| SEMA3D | grey |
| IRGC | grey |
| ARHGEF17 | grey |
| TEX101 | grey |
| CA12 | grey |
| LINC00672 | grey |
| MICAL2 | grey |
| PABPC5 | grey |
| FAM47E | grey |
| ZDHHC23 | grey |
| NET1 | grey |
| RXFP2 | grey |
| PCDH9 | grey |
| PRSS1 | grey |
| ATP10B | grey |
| GALNT14 | grey |
| BMX | grey |
| HOXA13 | grey |
| FREM3 | grey |
| PLPPR3 | grey |
| SESN2 | grey |
| LHCGR | grey |
| GABARAPL2 | grey |
| NPW | grey |
| VSIG1 | grey |
| UQCR11 | grey |
| EFCC1 | grey |
| ELL | grey |
| KRT2 | grey |
| CHI3L1 | grey |
| MUSK | grey |
| CD276 | grey |
| CAMK2A | grey |
| MPZ | grey |
| TRH | grey |
| TMEM54 | grey |
| SEMA6A | grey |
| FRRS1 | grey |
| CARD18 | grey |
| WDR93 | grey |
| PCDHGC5 | grey |
| TNFRSF1B | grey |
| CEACAM7 | grey |
| TREM1 | grey |
| EMILIN1 | grey |
| PFKFB3 | grey |
| HAS1 | grey |
| MTCL1 | grey |
| PI4K2A | grey |
| SLC27A6 | grey |
| AGMO | grey |
| JUNB | grey |
| STRIT1 | grey |
| KIFC3 | grey |
| CHGB | grey |
| PCDHB12 | grey |
| PLA2G2A | grey |
| DPP4 | grey |
| CCDC188 | grey |
| PILRB | grey |
| FER1L5 | grey |
| C1GALT1C1L | grey |
| ACAD11 | grey |
| ADRA2C | grey |
| CLECL1 | grey |
| C4orf19 | grey |
| SLC10A4 | grey |
| C1QTNF8 | grey |
| IL17RE | grey |
| GNB3 | grey |
| PPIP5K2 | grey |
| WNT10B | grey |
| GLRA2 | grey |
| FTH1 | grey |
| SOCS1 | grey |
| SPATA31E1 | grey |
| PCP4 | grey |
| KIF6 | grey |
| ACKR1 | grey |
| HTR3E | grey |
| SERTM2 | grey |
| CCDC8 | grey |
| SLC34A1 | grey |
| FGF11 | grey |
| LILRA4 | grey |
| FAM92A | grey |
| NHEJ1 | grey |
| ZNF521 | grey |
| PCED1B | grey |
| RERG | grey |
| ASPRV1 | grey |
| CLDN12 | grey |
| STPG4 | grey |
| SFTA2 | grey |
| SV2B | grey |
| ABHD12B | grey |
| RORB | grey |
| GOLGA7B | grey |
| ERF | grey |
| REG1B | grey |
| DAPL1 | grey |
| NKAIN4 | grey |
| NRN1 | grey |
| PDZRN4 | grey |
| FBP1 | grey |
| ADAMTS19 | grey |
| NPIPB9 | grey |
| FCN1 | grey |
| OR10A5 | grey |
| PIGZ | grey |
| MRAP | grey |
| GPR85 | grey |
| CA10 | grey |
| DMKN | grey |
| ZBED2 | grey |
| S100Z | grey |
| CT45A1 | grey |
| CLC | grey |
| ATP5F1E | grey |
| MUC7 | grey |
| GDPD4 | grey |
| MEST | grey |
| IVNS1ABP | grey |
| G0S2 | grey |
| USP41 | grey |
| PIH1D2 | grey |
| LRP4 | grey |
| CDKN1B | grey |
| CCDC169 | grey |
| FCAMR | grey |
| CXCL8 | grey |
| CROCC2 | grey |
| MAOA | grey |
| MAPRE3 | grey |
| PCDHGA9 | grey |
| RAB27B | grey |
| CDK3 | grey |
| TULP2 | grey |
| EFCAB8 | grey |
| PAPPA | grey |
| C3orf22 | grey |
| SPRR2G | grey |
| NOTUM | grey |
| PRB2 | grey |
| ANKRD37 | grey |
| AADAT | grey |
| GRIA2 | grey |
| MFAP2 | grey |
| DHRS11 | grey |
| CMKLR1 | grey |
| PMM1 | grey |
| CRYBA1 | grey |
| PKP1 | grey |
| SCUBE1 | grey |
| RUNDC3B | grey |
| MPZL3 | grey |
| STMN2 | grey |
| SYNPR | grey |
| SHANK1 | grey |
| ZNF385D | grey |
| IFIT1B | grey |
| HOXA9 | grey |
| MFAP5 | grey |
| PSMB9 | grey |
| RUNX1T1 | grey |
| SCGB2B2 | grey |
| HSPA1B | grey |
| FAM171A2 | grey |
| BAALC | grey |
| SPTLC3 | grey |
| ZNF843 | grey |
| MICALL2 | grey |
| C21orf62 | grey |
| HEY1 | grey |
| PROM1 | grey |
| SSTR5 | grey |
| NTNG1 | grey |
| AR | grey |
| AKR1B10 | grey |
| RUNDC3A | grey |
| BTG1 | grey |
| ADM5 | grey |
| GDF11 | grey |
| PPBP | grey |
| SLC47A2 | grey |
| NDUFA13 | grey |
| ANGPT1 | grey |
| HIST3H2A | grey |
| COL17A1 | grey |
| XCL1 | grey |
| NPBWR1 | grey |
| CASTOR1 | grey |
| MOGAT1 | grey |
| ACOT6 | grey |
| PPEF1 | grey |
| SMIM17 | grey |
| KNDC1 | grey |
| SV2A | grey |
| SLC10A2 | grey |
| CEACAM1 | grey |
| ZIM2 | grey |
| RANBP17 | grey |
| SCN2B | grey |
| KLRC1 | grey |
| COL5A1 | grey |
| C9orf153 | grey |
| DSG1 | grey |
| CYP7B1 | grey |
| CCDC168 | grey |
| ZNF415 | grey |
| CD177 | grey |
| TTLL7 | grey |
| TMEM272 | grey |
| MSLN | grey |
| SLC52A3 | grey |
| MXD1 | grey |
| UBC | grey |
| LRATD1 | grey |
| MMP12 | grey |
| BCKDHA | grey |
| HIST1H1C | grey |
| FCAR | grey |
| ZBTB46 | grey |
| PCSK9 | grey |
| CFAP77 | grey |
| CELA3B | grey |
| ATP6V0C | grey |
| MAP1LC3B | grey |
| SLC2A3 | grey |
| ARHGEF18 | grey |
| EGR4 | grey |
| LEPR | grey |
| MAB21L2 | grey |
| KLK7 | grey |
| GASK1A | grey |
| OPALIN | grey |
| ALOX15 | grey |
| MYL9 | grey |
| 8-Mar | grey |
| ABCA7 | grey |
| ENTPD3 | grey |
| LPCAT1 | grey |
| REC114 | grey |
| PGAM1 | grey |
| BMI1 | grey |
| DDIT4 | grey |
| FEZ1 | grey |
| HBZ | grey |
| TBC1D3F | grey |
| GPR45 | grey |
| TIGD3 | grey |
| ARHGAP44 | grey |
| CDH7 | grey |
| FAM110A | grey |
| SLC28A2 | grey |
| LAG3 | grey |
| CNNM1 | grey |
| OGDHL | grey |
| TONSL | grey |
| PSAP | grey |
| PSG9 | grey |
| PPP1R2C | grey |
| VANGL2 | grey |
| DNTT | grey |
| TGFBI | grey |
| UMODL1 | grey |
| IL7 | grey |
| MACC1 | grey |
| CCDC181 | grey |
| HBG2 | grey |
| ABHD17A | grey |
| OR52W1 | grey |
| ALDOA | grey |
| LYPD4 | grey |
| SERPINH1 | grey |
| PLP1 | grey |
| CNBD1 | grey |
| TEX45 | grey |
| OGN | grey |
| C9orf78 | grey |
| CSF2 | grey |
| LEKR1 | grey |
| NCR1 | grey |
| RETN | grey |
| PKIB | grey |
| ANGPTL1 | grey |
| SHISA4 | grey |
| KIR2DL4 | grey |
| TPSB2 | grey |
| DEFA1B | grey |
| TBC1D3L | grey |
| CNTNAP3 | grey |
| FAM209B | grey |
| AQP6 | grey |
| CABYR | grey |
| TEX14 | grey |
| NPR3 | grey |
| SMPDL3A | grey |
| CRYGD | grey |
| TMEM132D | grey |
| RAB3A | grey |
| CRIP1 | grey |
| EBF3 | grey |
| LRP1B | grey |
| PCDHA4 | grey |
| GNAO1 | grey |
| PAWR | grey |
| IL31RA | grey |
| DNM1 | grey |
| C1QTNF4 | grey |
| WLS | grey |
| CD274 | grey |
| CELA3A | grey |
| ATG2A | grey |
| IGFBP2 | grey |
| SUSD3 | grey |
| TMEM140 | grey |
| HNRNPUL2 | grey |
| SOCS3 | grey |
| TICAM2 | grey |
| PXDN | grey |
| DEFA5 | grey |
| HIST1H2AE | grey |
| TMEM150C | grey |
| FADS2 | grey |
| TMPRSS11F | grey |
| ZNF750 | grey |
| SULT1C2 | grey |
| STC1 | grey |
| DSG2 | grey |
| FRMD7 | grey |
| EPB42 | grey |
| STON1 | grey |
| SAMSN1 | grey |
| SSTR3 | grey |
| ARSA | grey |
| AGBL4 | grey |
| PROB1 | grey |
| PCDHB11 | grey |
| ZFY | grey |
| PCDHGA12 | grey |
| CD300E | grey |
| KIAA1211 | grey |
| PPP5D1 | grey |
| ARHGAP23 | grey |
| TMEM44 | grey |
| GDAP1L1 | grey |
| KRT4 | pink |
| SPINK5 | pink |
| KRT6C | pink |
| KRT6B | pink |
| CNFN | pink |
| PADI1 | pink |
| KRT78 | pink |
| TMPRSS11E | pink |
| KRT13 | pink |
| SPRR1A | pink |
| NCCRP1 | pink |
| IVL | pink |
| RHCG | pink |
| TMPRSS11B | pink |
| SPRR3 | pink |
| KRT24 | pink |
| SLURP1 | pink |
| CRCT1 | pink |
| MUC21 | pink |
| CRNN | pink |
| KLK6 | pink |
| SPRR2E | pink |
| SPRR2D | pink |
| SPRR2A | pink |
| KLK8 | pink |
| KRT6A | pink |
| LCK | red |
| SH2D1B | red |
| RUNX3 | red |
| S1PR5 | red |
| IL2RB | red |
| SPOCK2 | red |
| CD7 | red |
| PDZD4 | red |
| CD247 | red |
| CARD11 | red |
| ARL4C | red |
| NCR3 | red |
| PDE4A | red |
| LGR6 | red |
| STAT4 | red |
| IL21R | red |
| MVD | red |
| GZMB | red |
| SH2D2A | red |
| PPP2R2B | red |
| DTX3 | red |
| FBXO44 | red |
| ZAP70 | red |
| PLCH2 | red |
| CD3E | red |
| GZMM | red |
| MTFP1 | red |
| PLEKHF1 | red |
| FGFBP2 | red |
| DUSP2 | red |
| TIGIT | red |
| TBX21 | red |
| PRF1 | red |
| SKAP1 | red |
| SPON2 | red |
| PRSS23 | red |
| CLIC3 | red |
| KLRK1 | red |
| SEPTIN1 | red |
| FCRL6 | red |
| CCL4 | red |
| SAMD3 | red |
| EOMES | red |
| MMP23B | red |
| FASLG | red |
| TWSG1 | turquoise |
| TAS2R3 | turquoise |
| DGAT2L6 | turquoise |
| NPIPB8 | turquoise |
| NOSTRIN | turquoise |
| BRI3BP | turquoise |
| GUCY1A1 | turquoise |
| EAF2 | turquoise |
| RXFP4 | turquoise |
| ZNF285 | turquoise |
| STX19 | turquoise |
| AWAT1 | turquoise |
| ITPR2 | turquoise |
| ZNF492 | turquoise |
| ZNF154 | turquoise |
| ASPN | turquoise |
| FASTKD1 | turquoise |
| ATAD2 | turquoise |
| MSH2 | turquoise |
| ANKRD36 | turquoise |
| ZNF114 | turquoise |
| ZNF765 | turquoise |
| LRRC19 | turquoise |
| TMEM182 | turquoise |
| BUB1 | turquoise |
| NUP160 | turquoise |
| ARHGEF38 | turquoise |
| ZNF772 | turquoise |
| TMEM201 | turquoise |
| LRRC69 | turquoise |
| PCBD2 | turquoise |
| ATAD5 | turquoise |
| CUL5 | turquoise |
| LIN9 | turquoise |
| KRIT1 | turquoise |
| CDC7 | turquoise |
| ICA1L | turquoise |
| ZNF430 | turquoise |
| CA14 | turquoise |
| KIF9 | turquoise |
| KIF18B | turquoise |
| ZNF132 | turquoise |
| ANKRD36C | turquoise |
| PMM2 | turquoise |
| CIP2A | turquoise |
| ZNF91 | turquoise |
| RFC3 | turquoise |
| MTHFD1L | turquoise |
| WDR75 | turquoise |
| NAA25 | turquoise |
| MFSD4A | turquoise |
| CASP8AP2 | turquoise |
| CENPF | turquoise |
| IBTK | turquoise |
| DLG1 | turquoise |
| MTR | turquoise |
| ERVW-1 | turquoise |
| ATP6V0A2 | turquoise |
| EEF2K | turquoise |
| SYCP3 | turquoise |
| ZNF519 | turquoise |
| ZNF568 | turquoise |
| TRIO | turquoise |
| POLR3G | turquoise |
| RBP2 | turquoise |
| EEA1 | turquoise |
| MND1 | turquoise |
| FOXRED2 | turquoise |
| BCL11A | turquoise |
| CCN6 | turquoise |
| GLMN | turquoise |
| SPDYE1 | turquoise |
| BCOR | turquoise |
| STXBP4 | turquoise |
| TAS2R10 | turquoise |
| PABPC3 | turquoise |
| MYB | turquoise |
| C8orf37 | turquoise |
| ZNF670 | turquoise |
| ARL13A | turquoise |
| PUS7 | turquoise |
| ZFP62 | turquoise |
| ZNF572 | turquoise |
| MIB1 | turquoise |
| TATDN1 | turquoise |
| UBAP2 | turquoise |
| TFB2M | turquoise |
| IMMP1L | turquoise |
| ZNF507 | turquoise |
| CEP76 | turquoise |
| CCDC73 | turquoise |
| ZNF181 | turquoise |
| SAMD13 | turquoise |
| MIER3 | turquoise |
| HASPIN | turquoise |
| NAA15 | turquoise |
| PJVK | turquoise |
| SLC5A3 | turquoise |
| HNRNPA1L2 | turquoise |
| MYO9A | turquoise |
| ZNF484 | turquoise |
| ZNF829 | turquoise |
| ZNF724 | turquoise |
| NREP | turquoise |
| GVQW3 | turquoise |
| CCDC122 | turquoise |
| DEFB131B | turquoise |
| TUBGCP4 | turquoise |
| BIRC6 | turquoise |
| CPLANE1 | turquoise |
| WDR12 | turquoise |
| L3MBTL1 | turquoise |
| TAS2R5 | turquoise |
| NUDT13 | turquoise |
| ZC3H8 | turquoise |
| NHLRC2 | turquoise |
| ZNF529 | turquoise |
| CAAP1 | turquoise |
| RCCD1 | turquoise |
| MTRNR2L3 | turquoise |
| PM20D2 | turquoise |
| CEP57L1 | turquoise |
| TTF2 | turquoise |
| INCA1 | turquoise |
| DEPDC1B | turquoise |
| DEPDC4 | turquoise |
| SETMAR | turquoise |
| GPATCH11 | turquoise |
| SENP8 | turquoise |
| KIAA1549 | turquoise |
| ANKRD28 | turquoise |
| ZNF678 | turquoise |
| CCDC150 | turquoise |
| EFHC1 | turquoise |
| TOMM20L | turquoise |
| IPO7 | turquoise |
| ITGA4 | turquoise |
| ERCC8 | turquoise |
| SUV39H2 | turquoise |
| PFAS | turquoise |
| ZKSCAN3 | turquoise |
| SIPA1L3 | turquoise |
| USP46 | turquoise |
| NUTM2G | turquoise |
| ZNF302 | turquoise |
| NSD2 | turquoise |
| ZNF92 | turquoise |
| ZNF460 | turquoise |
| EFCAB10 | turquoise |
| TADA1 | turquoise |
| CATSPER2 | turquoise |
| CDK1 | turquoise |
| FANCM | turquoise |
| ZC3HAV1L | turquoise |
| UBE3D | turquoise |
| SMC2 | turquoise |
| ECM2 | turquoise |
| SPDYA | turquoise |
| SKA3 | turquoise |
| CCDC14 | turquoise |
| HEATR5B | turquoise |
| HACE1 | turquoise |
| ALG10B | turquoise |
| HSD17B12 | turquoise |
| CIT | turquoise |
| MBTPS2 | turquoise |
| SLX4IP | turquoise |
| DMXL1 | turquoise |
| ZNF195 | turquoise |
| DENND4C | turquoise |
| IFT81 | turquoise |
| ZNF439 | turquoise |
| KPNA5 | turquoise |
| DCAF17 | turquoise |
| PRIMPOL | turquoise |
| CNTLN | turquoise |
| ZNF730 | turquoise |
| EDDM13 | turquoise |
| ZBTB39 | turquoise |
| ZMYM4 | turquoise |
| ECT2 | turquoise |
| MTMR7 | turquoise |
| NCAPG | turquoise |
| PNPT1 | turquoise |
| STRBP | turquoise |
| NCAPG2 | turquoise |
| PIBF1 | turquoise |
| PRC1 | turquoise |
| GNPDA2 | turquoise |
| SACS | turquoise |
| ZBED3 | turquoise |
| UTP20 | turquoise |
| ZNF665 | turquoise |
| ERC1 | turquoise |
| WDR62 | turquoise |
| ZC3H12B | turquoise |
| EFCAB11 | turquoise |
| ALG10 | turquoise |
| MMP24 | turquoise |
| ETAA1 | turquoise |
| ATR | turquoise |
| ZNF518A | turquoise |
| MKI67 | turquoise |
| RAD54B | turquoise |
| LRP8 | turquoise |
| ANKRD61 | turquoise |
| POMK | turquoise |
| LARP1B | turquoise |
| NEK1 | turquoise |
| ZNF70 | turquoise |
| POU5F2 | turquoise |
| PUS10 | turquoise |
| ZNF544 | turquoise |
| MAT2A | turquoise |
| NUP155 | turquoise |
| ORC6 | turquoise |
| FBXO22 | turquoise |
| ZBED4 | turquoise |
| SOGA1 | turquoise |
| KIF11 | turquoise |
| KLB | turquoise |
| C2orf92 | turquoise |
| TMEM68 | turquoise |
| ERMP1 | turquoise |
| LNX1 | turquoise |
| ZNF708 | turquoise |
| ZSCAN31 | turquoise |
| ZNF850 | turquoise |
| ZNF551 | turquoise |
| SRSF10 | turquoise |
| ZNF593 | turquoise |
| VN1R1 | turquoise |
| DTWD1 | turquoise |
| ERCC5 | turquoise |
| MTRNR2L4 | turquoise |
| SPATA21 | turquoise |
| C4orf46 | turquoise |
| NKTR | turquoise |
| AHI1 | turquoise |
| CSNK2A3 | turquoise |
| CHD9 | turquoise |
| XPO4 | turquoise |
| RUFY2 | turquoise |
| KIAA1586 | turquoise |
| CFAP44 | turquoise |
| UBE3A | turquoise |
| DEPDC1 | turquoise |
| ZNF823 | turquoise |
| PAX6 | turquoise |
| MYO19 | turquoise |
| ZNF431 | turquoise |
| ZNF470 | turquoise |
| AQP11 | turquoise |
| PUS7L | turquoise |
| TAS2R46 | turquoise |
| PLET1 | turquoise |
| NUDT6 | turquoise |
| CEP192 | turquoise |
| ZRANB3 | turquoise |
| SELENOI | turquoise |
| LRRC8B | turquoise |
| BTBD18 | turquoise |
| GSTCD | turquoise |
| FANCB | turquoise |
| NPIPB4 | turquoise |
| KLHL23 | turquoise |
| CENPJ | turquoise |
| TRIM24 | turquoise |
| TIGD2 | turquoise |
| DEXI | turquoise |
| E2F8 | turquoise |
| ZNF813 | turquoise |
| DHX33 | turquoise |
| ZMAT1 | turquoise |
| TAF1A | turquoise |
| SLC35B4 | turquoise |
| ZNF583 | turquoise |
| LRRCC1 | turquoise |
| ANAPC1 | turquoise |
| PMS1 | turquoise |
| NOC3L | turquoise |
| POLQ | turquoise |
| RPAP2 | turquoise |
| MCM2 | turquoise |
| ZNF471 | turquoise |
| NEIL3 | turquoise |
| FAM161A | turquoise |
| TBC1D32 | turquoise |
| TOP2A | turquoise |
| FAN1 | turquoise |
| MLANA | turquoise |
| NAF1 | turquoise |
| ZNF573 | turquoise |
| TCAF1 | turquoise |
| NDUFAF6 | turquoise |
| RBM12B | turquoise |
| B4GALT6 | turquoise |
| BTBD3 | turquoise |
| C2CD2 | turquoise |
| ZACN | turquoise |
| KANK1 | turquoise |
| GVQW2 | turquoise |
| CCDC18 | turquoise |
| GPR179 | turquoise |
| ZNF382 | turquoise |
| MCM4 | turquoise |
| NEMP1 | turquoise |
| ZNF805 | turquoise |
| LATS1 | turquoise |
| TUBA3D | turquoise |
| TAS2R13 | turquoise |
| ANLN | turquoise |
| TMEM67 | turquoise |
| FAM227B | turquoise |
| IQCH | turquoise |
| NME9 | turquoise |
| ZSCAN20 | turquoise |
| WDR60 | turquoise |
| MRTFB | turquoise |
| USP54 | turquoise |
| AGPAT5 | turquoise |
| TTC3 | turquoise |
| TGFBRAP1 | turquoise |
| DCDC2B | turquoise |
| LUZP1 | turquoise |
| GPATCH8 | turquoise |
| PROX2 | turquoise |
| E2F7 | turquoise |
| GNL3L | turquoise |
| AGBL3 | turquoise |
| PACRGL | turquoise |
| RAD52 | turquoise |
| ZNF233 | turquoise |
| PPARA | turquoise |
| SPATA25 | turquoise |
| KCTD4 | turquoise |
| METAP1D | turquoise |
| TFEC | turquoise |
| GOLGA8H | turquoise |
| ACP4 | turquoise |
| KATNAL2 | turquoise |
| MELK | turquoise |
| CKAP2L | turquoise |
| MANEA | turquoise |
| GOLGA8K | turquoise |
| MTHFD2L | turquoise |
| TUBGCP5 | turquoise |
| UBAP1L | turquoise |
| CCNJ | turquoise |
| PTGR2 | turquoise |
| PAXIP1 | turquoise |
| GPR75 | turquoise |
| TMEM262 | turquoise |
| NCAPD3 | turquoise |
| SLC24A1 | turquoise |
| POGLUT3 | turquoise |
| CENPE | turquoise |
| TAS2R4 | turquoise |
| IFT74 | turquoise |
| TCERG1 | turquoise |
| ASB14 | turquoise |
| DISP2 | turquoise |
| SOCS7 | turquoise |
| TAS2R50 | turquoise |
| SPATA7 | turquoise |
| DOCK7 | turquoise |
| MCM10 | turquoise |
| RNASEH2B | turquoise |
| SLFNL1 | turquoise |
| PYURF | turquoise |
| NADK2 | turquoise |
| APPL1 | turquoise |
| WDR43 | turquoise |
| FER | turquoise |
| BRCA1 | turquoise |
| SLC9A7 | turquoise |
| C11orf74 | turquoise |
| SGO1 | turquoise |
| ZNF846 | turquoise |
| ZNF138 | turquoise |
| RUNX1 | turquoise |
| TARBP1 | turquoise |
| ZNF543 | turquoise |
| ZNF260 | turquoise |
| MIGA1 | turquoise |
| CEP295 | turquoise |
| LCTL | turquoise |
| SLC12A2 | turquoise |
| ZNF268 | turquoise |
| PDC | turquoise |
| CETN3 | turquoise |
| CCDC138 | turquoise |
| PCLAF | turquoise |
| GTF3C4 | turquoise |
| TTBK2 | turquoise |
| ZNF610 | turquoise |
| KIF20A | turquoise |
| POLR1A | turquoise |
| BICD1 | turquoise |
| CCAR1 | turquoise |
| TYMS | turquoise |
| PLCE1 | turquoise |
| HLTF | turquoise |
| ERI2 | turquoise |
| FOXM1 | turquoise |
| PMFBP1 | turquoise |
| HTR2B | turquoise |
| KNL1 | turquoise |
| ERICH6 | turquoise |
| SLC25A15 | turquoise |
| MARS2 | turquoise |
| OTUD4 | turquoise |
| BRIP1 | turquoise |
| RBM41 | turquoise |
| ECT2L | turquoise |
| SRFBP1 | turquoise |
| NIPSNAP3B | turquoise |
| FKTN | turquoise |
| PLEKHA5 | turquoise |
| ZNF280D | turquoise |
| MGA | turquoise |
| SASS6 | turquoise |
| SPN | turquoise |
| YY2 | turquoise |
| ZSCAN5A | turquoise |
| DTD2 | turquoise |
| ZNF221 | turquoise |
| ICE2 | turquoise |
| RAVER2 | turquoise |
| SPIN2A | turquoise |
| ZFP2 | turquoise |
| VPS13A | turquoise |
| PAXBP1 | turquoise |
| ZNF569 | turquoise |
| FIGNL1 | turquoise |
| ESPL1 | turquoise |
| PRPF40B | turquoise |
| FAM217A | turquoise |
| RAD54L | turquoise |
| ZNF559-ZNF177 | turquoise |
| ENOSF1 | turquoise |
| MAN2A1 | turquoise |
| TBCK | turquoise |
| LRRC1 | turquoise |
| APLF | turquoise |
| DTWD2 | turquoise |
| LRRC58 | turquoise |
| SLC35A3 | turquoise |
| ZNF367 | turquoise |
| SLC7A1 | turquoise |
| CENPK | turquoise |
| ZNF93 | turquoise |
| C20orf194 | turquoise |
| SPDYE5 | turquoise |
| BCL2L2-PABPN1 | turquoise |
| MEIOC | turquoise |
| SLC3A1 | turquoise |
| TCTN2 | turquoise |
| TAF4B | turquoise |
| SHPRH | turquoise |
| TIAF1 | turquoise |
| LACTB2 | turquoise |
| REXO5 | turquoise |
| PKD2L2 | turquoise |
| EHBP1 | turquoise |
| TRPM7 | turquoise |
| PRIM1 | turquoise |
| SH3RF1 | turquoise |
| DNAJB7 | turquoise |
| ZGRF1 | turquoise |
| NEK5 | turquoise |
| C1QTNF3 | turquoise |
| DUS4L | turquoise |
| CENPN | turquoise |
| ARL2BP | turquoise |
| MAP4K5 | turquoise |
| SMC4 | turquoise |
| LARP4 | turquoise |
| B3GALNT2 | turquoise |
| FANCI | turquoise |
| GPR135 | turquoise |
| ZNF491 | turquoise |
| UCK2 | turquoise |
| AKAP11 | turquoise |
| BRCA2 | turquoise |
| UBN2 | turquoise |
| DDX47 | turquoise |
| CEP83 | turquoise |
| GRK4 | turquoise |
| SRR | turquoise |
| IFT80 | turquoise |
| USP51 | turquoise |
| LRP12 | turquoise |
| TAC4 | turquoise |
| KRR1 | turquoise |
| CEP128 | turquoise |
| SOCS6 | turquoise |
| THAP9 | turquoise |
| SCML2 | turquoise |
| AKAP9 | turquoise |
| ZNF717 | turquoise |
| THOC2 | turquoise |
| ACSBG2 | turquoise |
| PCNX4 | turquoise |
| TAS2R31 | turquoise |
| CHAMP1 | turquoise |
| CNTRL | turquoise |
| TMTC3 | turquoise |
| ZNF790 | turquoise |
| AMER1 | turquoise |
| GPAM | turquoise |
| ZNF320 | turquoise |
| TRUB1 | turquoise |
| KCNJ13 | turquoise |
| TMEM17 | turquoise |
| C1QL3 | turquoise |
| MAD2L1 | turquoise |
| ZNF451 | turquoise |
| TTK | turquoise |
| MDN1 | turquoise |
| CCDC58 | turquoise |
| SLC25A36 | turquoise |
| MSH6 | turquoise |
| PLA2G10 | turquoise |
| KNOP1 | turquoise |
| CARF | turquoise |
| TTC30A | turquoise |
| NUP35 | turquoise |
| ZNF808 | turquoise |
| CD3EAP | turquoise |
| PRDM11 | turquoise |
| ZMYM1 | turquoise |
| KCNIP4 | turquoise |
| ZNF337 | turquoise |
| KIF24 | turquoise |
| SLC13A4 | turquoise |
| ZNF146 | turquoise |
| ZNF248 | turquoise |
| CENPI | turquoise |
| FANCA | turquoise |
| TMPPE | turquoise |
| DNA2 | turquoise |
| ZNF37A | turquoise |
| ALG11 | turquoise |
| GINS1 | turquoise |
| MEMO1 | turquoise |
| ZNF124 | turquoise |
| PRR11 | turquoise |
| MTBP | turquoise |
| SGO2 | turquoise |
| MACF1 | turquoise |
| RABGGTB | turquoise |
| VPS13C | turquoise |
| ACACA | turquoise |
| STIL | turquoise |
| C4orf36 | turquoise |
| SSBP2 | turquoise |
| ZNF347 | turquoise |
| ZNF675 | turquoise |
| TMEM97 | turquoise |
| AFG1L | turquoise |
| SPIN1 | turquoise |
| RPGRIP1L | turquoise |
| ARID1B | turquoise |
| PRELID2 | turquoise |
| SKP2 | turquoise |
| CTDSPL2 | turquoise |
| ZNF891 | turquoise |
| GTF2I | turquoise |
| COX20 | turquoise |
| ZNF621 | turquoise |
| UHRF1 | turquoise |
| ERG | turquoise |
| ZNF660 | turquoise |
| ARFGEF2 | turquoise |
| CDC25A | turquoise |
| EPDR1 | turquoise |
| RSKR | turquoise |
| ZNF410 | turquoise |
| FANCL | turquoise |
| FAM120C | turquoise |
| ASH1L | turquoise |
| WDR31 | turquoise |
| ZNF345 | turquoise |
| DCLRE1A | turquoise |
| POLR1B | turquoise |
| ZNF22 | turquoise |
| CHKB | turquoise |
| CENPQ | turquoise |
| KIF20B | turquoise |
| HESX1 | turquoise |
| CEP85 | turquoise |
| ZNF749 | turquoise |
| PGBD1 | turquoise |
| USP37 | turquoise |
| USP34 | turquoise |
| NUBPL | turquoise |
| WDR88 | turquoise |
| PHF6 | turquoise |
| FRMD5 | turquoise |
| TMEM266 | turquoise |
| MYCBP2 | turquoise |
| ZNF566 | turquoise |
| ZNF607 | turquoise |
| CERS6 | turquoise |
| PPFIBP1 | turquoise |
| ZNF761 | turquoise |
| SPIN4 | turquoise |
| ZNF681 | turquoise |
| ZNF629 | turquoise |
| IQGAP3 | turquoise |
| RPP40 | turquoise |
| TRMT11 | turquoise |
| BDP1 | turquoise |
| CENPP | turquoise |
| HSPA14 | turquoise |
| C20orf144 | turquoise |
| URB1 | turquoise |
| ZNF714 | turquoise |
| WRN | turquoise |
| ZNF567 | turquoise |
| ZNF577 | turquoise |
| CATSPERE | turquoise |
| RAD50 | turquoise |
| U2SURP | turquoise |
| PDE6C | turquoise |
| ALDH18A1 | turquoise |
| TAS2R20 | turquoise |
| ZFP14 | turquoise |
| PDE8B | turquoise |
| DARS2 | turquoise |
| SPDYE3 | turquoise |
| CCNA2 | turquoise |
| ZNF43 | turquoise |
| ZNF283 | turquoise |
| ZNF100 | turquoise |
| ZNF649 | turquoise |
| PSTK | turquoise |
| TSTD2 | turquoise |
| TMEM267 | turquoise |
| KLHL42 | turquoise |
| KCNQ5 | turquoise |
| DNM1L | turquoise |
| POLG2 | turquoise |
| CSPP1 | turquoise |
| BARD1 | turquoise |
| TRPC5 | turquoise |
| MPHOSPH9 | turquoise |
| LYRM7 | turquoise |
| DTL | turquoise |
| CCDC30 | turquoise |
| ZNF565 | turquoise |
| GOLGA8N | turquoise |
| MAP4K3 | turquoise |
| MBLAC2 | turquoise |
| MLLT10 | turquoise |
| KNTC1 | turquoise |
| ZNF121 | turquoise |
| IFT172 | turquoise |
| MCM8 | turquoise |
| POLE2 | turquoise |
| FBXL2 | turquoise |
| L2HGDH | turquoise |
| WDPCP | turquoise |
| C18orf54 | turquoise |
| CEP41 | turquoise |
| TIMM23B | turquoise |
| PHF14 | turquoise |
| ZNF726 | turquoise |
| CEP135 | turquoise |
| GINS3 | turquoise |
| TAS2R14 | turquoise |
| WEE1 | turquoise |
| TIPIN | turquoise |
| MIS18BP1 | turquoise |
| ANKRD50 | turquoise |
| EFCAB5 | turquoise |
| KAT6B | turquoise |
| ANKRD26 | turquoise |
| ERCC6L | turquoise |
| CYB5D1 | turquoise |
| HEATR4 | turquoise |
| CYP20A1 | turquoise |
| RAD51AP1 | turquoise |
| ADGRA3 | turquoise |
| HMMR | turquoise |
| CDCA7 | turquoise |
| KLHDC1 | turquoise |
| PIGW | turquoise |
| WDHD1 | turquoise |
| FKBP7 | turquoise |
| CNST | turquoise |
| CKAP2 | turquoise |
| FANCD2OS | turquoise |
| ZNF497 | turquoise |
| LRRTM2 | turquoise |
| SLC39A10 | turquoise |
| MFSD4B | turquoise |
| MORN1 | turquoise |
| ACRV1 | turquoise |
| LRRC34 | turquoise |
| TMEM209 | turquoise |
| BUB1B | turquoise |
| KCTD3 | turquoise |
| HPS4 | turquoise |
| ZNF440 | turquoise |
| REV3L | turquoise |
| ZKSCAN2 | turquoise |
| SERHL2 | turquoise |
| ATP8B1 | turquoise |
| ZNF141 | turquoise |
| RGS20 | turquoise |
| TEC | turquoise |
| MSRB3 | turquoise |
| NFYB | turquoise |
| PHOSPHO2 | turquoise |
| PARPBP | turquoise |
| TRNT1 | turquoise |
| ZFAND1 | turquoise |
| TSSK4 | turquoise |
| TMTC4 | turquoise |
| NR2C1 | turquoise |
| TOGARAM1 | turquoise |
| CCDC200 | turquoise |
| RNF212B | turquoise |
| GEN1 | turquoise |
| BCKDHB | turquoise |
| TAS2R19 | turquoise |
| TMEM106A | turquoise |
| TTC19 | turquoise |
| ZNF69 | turquoise |
| DPY19L4 | turquoise |
| ACER2 | turquoise |
| C17orf78 | turquoise |
| TSEN2 | turquoise |
| FAM186B | turquoise |
| THNSL1 | turquoise |
| C12orf66 | turquoise |
| ZBTB41 | turquoise |
| ZNF280C | turquoise |
| RBM26 | turquoise |
| CLHC1 | turquoise |
| LDHAL6A | turquoise |
| DNAJC2 | turquoise |
| SEH1L | turquoise |
| KANSL1L | turquoise |
| FBXO43 | turquoise |
| HERC2 | turquoise |
| RBM34 | turquoise |
| ZNF41 | turquoise |
| CENPC | turquoise |
| MAST4 | turquoise |
| TIGD1 | turquoise |
| POLA1 | turquoise |
| KCNV2 | turquoise |
| PKD2 | turquoise |
| ZBED6 | turquoise |
| SPC25 | turquoise |
| SLC15A2 | turquoise |
| APOOL | turquoise |
| ESCO2 | turquoise |
| RCN1 | turquoise |
| PAICS | turquoise |
| INTS7 | turquoise |
| VEZT | turquoise |
| SLC35G6 | turquoise |
| CDCA2 | turquoise |
| ZDHHC21 | turquoise |
| URB2 | turquoise |
| FAM184B | turquoise |
| ZNF680 | turquoise |
| ZNF280B | turquoise |
| TFDP2 | turquoise |
| ERCC4 | turquoise |
| ZNF677 | turquoise |
| METTL8 | turquoise |
| GPSM2 | turquoise |
| ZNF618 | turquoise |
| CEP290 | turquoise |
| ZNF528 | turquoise |
| MRPL42 | turquoise |
| CHMP4A | turquoise |
| EXO1 | turquoise |
| ZNF562 | turquoise |
| HIGD1B | turquoise |
| MTAP | turquoise |
| TEX30 | turquoise |
| PP2D1 | turquoise |
| LRPPRC | turquoise |
| JADE3 | turquoise |
| C5orf24 | turquoise |
| CBX5 | turquoise |
| FUT10 | turquoise |
| ZNF326 | turquoise |
| SMARCAD1 | turquoise |
| S100G | turquoise |
| ADAT2 | turquoise |
| ANKAR | turquoise |
| SLC26A2 | turquoise |
| MIS18A | turquoise |
| TMEM220 | turquoise |
| CREB3L2 | turquoise |
| TEX38 | turquoise |
| CLSPN | turquoise |
| SNX32 | turquoise |
| NARS2 | turquoise |
| KIF15 | turquoise |
| TM7SF3 | turquoise |
| IFT140 | turquoise |
| SETDB2 | turquoise |
| IKZF4 | turquoise |
| CHEK1 | turquoise |
| ZNF594 | turquoise |
| ATP8B4 | turquoise |
| PAQR3 | turquoise |
| RRP15 | turquoise |
| EFCAB13 | turquoise |
| ZWILCH | turquoise |
| B3GALNT1 | turquoise |
| TCF12 | turquoise |
| PDS5A | turquoise |
| TRMT13 | turquoise |
| MAP7D3 | turquoise |
| RREB1 | turquoise |
| USP45 | turquoise |
| ZNF85 | turquoise |
| INTS2 | turquoise |
| SPDL1 | turquoise |
| AGO3 | turquoise |
| CCNE2 | turquoise |
| PRH2 | turquoise |
| RHBDL2 | turquoise |
| CCDC15 | turquoise |
| CENPU | turquoise |
| PIGM | turquoise |
| CASD1 | turquoise |
| PLK4 | turquoise |
| DRC3 | turquoise |
| HMBOX1 | turquoise |
| SCMH1 | turquoise |
| C1orf146 | turquoise |
| ZKSCAN8 | turquoise |
| GSE1 | turquoise |
| OIP5 | turquoise |
| SFXN2 | turquoise |
| ESRP2 | turquoise |
| PTCD2 | turquoise |
| CEP55 | turquoise |
| PRMT3 | turquoise |
| RAB9B | turquoise |
| ABCE1 | turquoise |
| ALMS1 | turquoise |
| HEATR1 | turquoise |
| RBL1 | turquoise |
| CCDC88A | turquoise |
| ZNF878 | turquoise |
| EYS | turquoise |
| XRCC2 | turquoise |
| ARR3 | turquoise |
| BRMS1L | turquoise |
| CCDC38 | turquoise |
| PLAGL1 | turquoise |
| BPTF | turquoise |
| C12orf71 | turquoise |
| THOC1 | turquoise |
| BACE1 | turquoise |
| ACYP1 | turquoise |
| SPDYE2 | turquoise |
| ITGB3BP | turquoise |
| CLASP2 | turquoise |
| ZFP28 | turquoise |
| GOLIM4 | turquoise |
| TEX10 | turquoise |
| DNAL1 | turquoise |
| KATNAL1 | turquoise |
| ZNF550 | turquoise |
| ZNF84 | turquoise |
| ZNF736 | turquoise |
| FAM135A | turquoise |
| CLOCK | turquoise |
| N4BP2 | turquoise |
| OCLM | turquoise |
| ZSCAN12 | turquoise |
| PDIK1L | turquoise |
| IRAK1BP1 | turquoise |
| MATR3 | turquoise |
| ZNF28 | turquoise |
| HTD2 | turquoise |
| RAD9B | turquoise |
| OCRL | turquoise |
| EIF2AK4 | turquoise |
| TBC1D24 | turquoise |
| MMS22L | turquoise |
| C3orf49 | turquoise |
| SEPTIN11 | turquoise |
| ASPM | turquoise |
| ZFP82 | turquoise |
| SREK1 | turquoise |
| LYG2 | turquoise |
| ZNF26 | turquoise |
| DNAJC24 | turquoise |
| WDR27 | turquoise |
| BRIX1 | turquoise |
| KIF23 | turquoise |
| FAM216A | turquoise |
| CDHR5 | turquoise |
| ZNF433 | turquoise |
| ZNF782 | turquoise |
| TMEM237 | turquoise |
| ANKRD36B | turquoise |
| ATRX | turquoise |
| GNRHR | turquoise |
| QSER1 | turquoise |
| CDC45 | turquoise |
| BEND3 | turquoise |
| KIF18A | turquoise |
| NKIRAS1 | turquoise |
| HACD3 | turquoise |
| AK6 | turquoise |
| ABL2 | turquoise |
| PANK1 | turquoise |
| GNPNAT1 | turquoise |
| SPICE1 | turquoise |
| GAN | turquoise |
| PIKFYVE | turquoise |
| GREB1L | turquoise |
| EME1 | turquoise |
| ZMYND8 | turquoise |
| ZNF721 | turquoise |
| ZNF253 | turquoise |
| DSCC1 | turquoise |
| TRIM45 | turquoise |
| MKRN2OS | turquoise |
| NUF2 | turquoise |
| RAI1 | turquoise |
| ZNF546 | turquoise |
| NDC1 | turquoise |
| ZNF514 | turquoise |
| TSPYL6 | turquoise |
| RCN2 | turquoise |
| KLHL11 | turquoise |
| WNT2B | turquoise |
| C5orf34 | turquoise |
| TEX9 | turquoise |
| TMA16 | turquoise |
| ITPR1 | turquoise |
| PPAT | turquoise |
| C1orf112 | turquoise |
| P2RY4 | turquoise |
| ZFP30 | turquoise |
| ZNF66 | turquoise |
| ZNF609 | turquoise |
| ESF1 | turquoise |
| KIF14 | turquoise |
| ZMYM3 | turquoise |
| ZNF605 | turquoise |
| EZH2 | turquoise |
| RFX7 | turquoise |
| STKLD1 | turquoise |
| KMT2A | turquoise |
| ZHX1 | turquoise |
| OSGEPL1 | turquoise |
| SCAI | turquoise |
| NRIP2 | turquoise |
| SCARB1 | turquoise |
| RIF1 | turquoise |
| ARHGAP11A | turquoise |
| CEP152 | turquoise |
| CCDC171 | turquoise |
| ZNF682 | turquoise |
| CTPS2 | turquoise |
| ZNF461 | turquoise |
| HELLS | turquoise |
| PFN4 | turquoise |
| GPR180 | turquoise |
| RAB40A | turquoise |
| DYNC2LI1 | turquoise |
| UHRF1BP1 | turquoise |
| ZNF644 | turquoise |
| TYW3 | turquoise |
| DOP1A | turquoise |
| PBRM1 | turquoise |
| TICRR | turquoise |
| INO80D | turquoise |
| VMA21 | turquoise |
| DIAPH3 | turquoise |
| NUDCD1 | turquoise |
| DNAAF2 | turquoise |
| WDR73 | turquoise |
| NEMF | turquoise |
| EXTL2 | turquoise |
| PPARGC1B | turquoise |
| OTUD6B | turquoise |
| EBLN2 | turquoise |
| TASOR2 | turquoise |
| EOGT | turquoise |
| ZC3H11A | turquoise |
| CDK6 | turquoise |
| ZNF737 | turquoise |
| MNS1 | turquoise |
| USP13 | turquoise |
| SMIM8 | turquoise |
| LCA5L | turquoise |
| MTFR1 | turquoise |
| C20orf96 | turquoise |
| ZNF501 | turquoise |
| ZNF236 | turquoise |
| PLCB1 | turquoise |
| RAB40AL | turquoise |
| TRIP13 | turquoise |
| ATP5MGL | turquoise |
| ZNF852 | turquoise |
| NBEAL1 | turquoise |
| CARNMT1 | turquoise |
| ZNF713 | turquoise |
| CLDN20 | turquoise |
| MYO1H | turquoise |
| A1CF | yellow |
| MAT1A | yellow |
| UGT1A1 | yellow |
| AFM | yellow |
| ADH1C | yellow |
| C8B | yellow |
| SLC2A2 | yellow |
| SLC17A4 | yellow |
| GSTA2 | yellow |
| SAA4 | yellow |
| CCDC196 | yellow |
| TM4SF4 | yellow |
| HNF4A | yellow |
| TTR | yellow |
| BHMT2 | yellow |
| SERPINA11 | yellow |
| SULT2A1 | yellow |
| NUPR1 | yellow |
| DHRS2 | yellow |
| UGT2B15 | yellow |
| MOGAT2 | yellow |
| SPINK1 | yellow |
| TAT | yellow |
| PCK1 | yellow |
| SERPINA10 | yellow |
| AMBP | yellow |
| AHSG | yellow |
| SLCO1B1 | yellow |
| HAO1 | yellow |
| HPR | yellow |
| HAO2 | yellow |
| CPN2 | yellow |
| UGT2B7 | yellow |
| GJB1 | yellow |
| DIO1 | yellow |
| CYP4A11 | yellow |
| ACSM2B | yellow |
| C4BPB | yellow |
| CYP2C19 | yellow |
| FABP1 | yellow |
| AGXT | yellow |
| HABP2 | yellow |
| ORM1 | yellow |
| HOGA1 | yellow |
| ITIH1 | yellow |
| CFHR2 | yellow |
| SHBG | yellow |
| HJV | yellow |
| FGF21 | yellow |
| ALDH1L1 | yellow |
| HPD | yellow |
| SPP2 | yellow |
| BAAT | yellow |
| APOA1 | yellow |
| BHMT | yellow |
| HMGCS2 | yellow |
| HPX | yellow |
| CLRN3 | yellow |
| ORM2 | yellow |
| AKR1D1 | yellow |
| CCL15 | yellow |
| AADAC | yellow |
| MASP1 | yellow |
| APOC3 | yellow |
| APOC2 | yellow |
| APOH | yellow |
| CFHR4 | yellow |
| FGB | yellow |
| ACSM2A | yellow |
| KNG1 | yellow |
| ALB | yellow |
| LECT2 | yellow |
| GRB14 | yellow |
| SERPINA3 | yellow |
| GOLT1A | yellow |
| ADH4 | yellow |
| CYP2C9 | yellow |
| PRODH2 | yellow |
| CPN1 | yellow |
| HGD | yellow |
| CFHR1 | yellow |
| INS-IGF2 | yellow |
| SLC28A1 | yellow |
| NR1H4 | yellow |
| ADH1B | yellow |
| OTC | yellow |
| APOA5 | yellow |
| ADH1A | yellow |
| GLYAT | yellow |
| UGT2B4 | yellow |
| TM4SF5 | yellow |
| SERPINA5 | yellow |
| FGL1 | yellow |
| APOC4 | yellow |
| CFHR5 | yellow |
| DAO | yellow |
| CCL16 | yellow |
| NR0B2 | yellow |
| DPYS | yellow |
| FGA | yellow |
| AKR1C4 | yellow |
| C1orf115 | yellow |
| PON1 | yellow |
| SERPINC1 | yellow |
| SERPINA4 | yellow |
| SERPINA6 | yellow |
| FGG | yellow |
| ADRA1A | yellow |
| F9 | yellow |
| PLG | yellow |
| APCS | yellow |
| MBL2 | yellow |
| APOC1 | yellow |
| RBP4 | yellow |
| SERPINA7 | yellow |
| CYP3A4 | yellow |
| ANG | yellow |
| AZGP1 | yellow |
| HRG | yellow |
| APOA2 | yellow |
| FETUB | yellow |
| IGFBP1 | yellow |
| GC | yellow |
| ALDOB | yellow |
| CYP2C18 | yellow |
| C6 | yellow |
| PAH | yellow |
| EVA1A | yellow |
| CYP1A2 | yellow |
| C8A | yellow |
