# Supplementary material for: Identification of hub genes associated with adult acute myeloid leukemia progression through weighted gene co-expression network analysis
Source: Aging (Albany NY). 2021 Feb 11;13(4):5686–97. doi: 10.18632/aging.202493 (PMC7950274; doi:10.18632/aging.202493)
Supplement: Supplementary Table 2 [file aging-13-202493-s002.pdf]

SUPPLEMENTARY TABLE

Supplementary Table 2. TFs and ncRNAs related to blue and turquoise modules.

module	ncRNA	connection	pvalue
blue	AC058791	56	0.000861
blue	AFAP1-AS1	590	5.31E-06
blue	ANCR	795	1.31E-07
blue	BANCR	311	0.003088
blue	CASC15	120	0.000202
blue	CISTR	2883	2.80E-18
blue	CRNDE	1984	6.75E-14
blue	FBXL19-AS1	30	0.007103
blue	FENDRR	2672	1.39E-28
blue	FMR1-AS1	619	3.66E-06
blue	GAS5	1067	0.000113
blue	LINC00673	157	0.005009
blue	LINC01242	24	0.003754
blue	MALAT1	1569	1.04E-10
blue	MEG3	12	0.000459
blue	MIR17HG	800	4.69E-07
blue	NORAD	1028	0.001439
blue	NRAV	622	1.91E-09
blue	NRCP	221	4.35E-05
blue	RAD51-AS1	905	7.89E-08
blue	RP5-1039K5	27	0.005269
blue	SBF2-AS1	275	0.000336
blue	SLC25A25-AS1	169	0.00741
blue	SNHG16	723	0.003953
blue	TFAP2A-AS2	620	0.00071
turquoise	AC005618.1	4	0.004255
turquoise	AC007563	5	0.006965
turquoise	AC012593	4	0.004255
turquoise	AQP4-AS1	5	0.006965
turquoise	BANCR	311	0.000969
turquoise	CISTR	2883	3.52E-14
turquoise	CRNDE	1984	6.87E-25
turquoise	CTA-212A2	30	0.001183
turquoise	CTD-3099C6	4	0.004255
turquoise	DLX6-AS1	14	0.005798
turquoise	DNAJC27-AS1	4	0.004255
turquoise	DRAIC	386	5.07E-11
turquoise	FENDRR	2672	2.57E-18
turquoise	FMR1-AS1	619	5.27E-16
turquoise	GAS5	1067	5.20E-09
turquoise	HELLPAR	138	0.000106
turquoise	LINC01247	3	0.002167
turquoise	LOC100996455	3	0.002167
turquoise	LOC101927450	3	0.002167
turquoise	LOC101927497	237	1.12E-13

turquoise	LOC101928404	3	0.002167
turquoise	MALAT1	1569	3.33E-35
turquoise	MIR17HG	800	4.93E-05
turquoise	MIR663AHG	15	0.007103
turquoise	NORAD	1028	1.83E-05
turquoise	NRCP	221	0.000128
turquoise	RAD51-AS1	905	2.39E-12
turquoise	AC103702.1	65	0.001888
turquoise	RP11-438D14	2	0.000736
turquoise	RP11-539I5	2	0.000736
turquoise	RP11-96C23	3	0.002167
turquoise	RP4-669H2	3	0.002167
turquoise	RP5-1039K5	27	0.000717
turquoise	SBF2-AS1	275	2.84E-09
turquoise	SENCR	123	0.006505
turquoise	SNHG16	723	4.00E-11
turquoise	STXBP5-AS1	284	1.27E-05
turquoise	TFAP2A-AS2	620	1.50E-23
turquoise	TRG-AS1	2	0.000736
turquoise	TUG1	228	8.76E-22
